# PT-Flax (phenotyping and TILLinG of flax): development of a flax (*Linum usitatissimum* L.) mutant population and TILLinG platform for forward and reverse genetics

**DOI:** 10.1186/1471-2229-13-159

**Published:** 2013-10-15

**Authors:** Maxime Chantreau, Sébastien Grec, Laurent Gutierrez, Marion Dalmais, Christophe Pineau, Hervé Demailly, Christine Paysant-Leroux, Reynald Tavernier, Jean-Paul Trouvé, Manash Chatterjee, Xavier Guillot, Véronique Brunaud, Brigitte Chabbert, Olivier van Wuytswinkel, Abdelhafid Bendahmane, Brigitte Thomasset, Simon Hawkins

**Affiliations:** 1 Université Lille Nord de France, Lille 1 UMR 1281, Villeneuve d'Ascq cedex F-59650, France; 2 INRA UMR, 281 Stress Abiotiques et Différenciation des Végétaux Cultivés, Villeneuve d’Ascq F-59650, France; 3 CRRBM, UFR des Sciences, UPJV, 33 rue Saint Leu, Amiens cedex 80039, France; 4 URGV, Unité de Recherche en Génomique Végétale, Université d'Evry Val d'Essonne, INRA, 2 rue Gaston Crémieux CP 5708, Evry cedex 91057, France; 5 LINEA, 20 Avenue Saget, Grandvilliers, 60 210, France; 6 Terre de Lin, société cooperative agricole, Saint-Pierre-Le-Viger, 76 740, France; 7 Bench Bio Pvt Ltd., c/o Jai Research Foundation, Vapi, Gujarat 396195, India; 8 National University of Ireland Galway (NUIG), University Road, Galway, Ireland; 9 Laboulet Semences, Airaines, 80 270, France; 10 INRA, UMR614 Fractionnement des AgroRessources et Environnement, Reims F-51100, France; 11 Université de Reims Champagne-Ardenne, UMR614 Fractionnement des AgroRessources et Environnement, Reims F-51100, France; 12 EA 3900-BioPI, UFR des Sciences, UPJV, 33 rue Saint Leu, Amiens cedex 80039, France; 13 CNRS-FRE 3580, GEC, Université de Technologie de Compiègne, CS 60319, Compiègnecedex 60203, France

**Keywords:** Flax, TILLinG, Mutants, Fiber, Lignin, Lignan, Oil, Fatty acids

## Abstract

**Background:**

Flax (*Linum usitatissimum* L.) is an economically important fiber and oil crop that has been grown for thousands of years. The genome has been recently sequenced and transcriptomics are providing information on candidate genes potentially related to agronomically-important traits. In order to accelerate functional characterization of these genes we have generated a flax EMS mutant population that can be used as a TILLinG (Targeting Induced Local Lesions in Genomes) platform for forward and reverse genetics.

**Results:**

A population of 4,894 M2 mutant seed families was generated using 3 different EMS concentrations (0.3%, 0.6% and 0.75%) and used to produce M2 plants for subsequent phenotyping and DNA extraction. 10,839 viable M2 plants (4,033 families) were obtained and 1,552 families (38.5%) showed a visual developmental phenotype (stem size and diameter, plant architecture, flower-related). The majority of these families showed more than one phenotype. Mutant phenotype data are organised in a database and can be accessed and searched at UTILLdb (http://urgv.evry.inra.fr/UTILLdb). Preliminary screens were also performed for atypical fiber and seed phenotypes. Genomic DNA was extracted from 3,515 M2 families and eight-fold pooled for subsequent mutant detection by ENDO1 nuclease mis-match cleavage. In order to validate the collection for reverse genetics, DNA pools were screened for two genes coding enzymes of the lignin biosynthesis pathway: *Coumarate-3-Hydroxylase* (*C3H*) and *Cinnamyl Alcohol Dehydrogenase* (*CAD*). We identified 79 and 76 mutations in the *C3H* and *CAD* genes, respectively. The average mutation rate was calculated as 1/41 Kb giving rise to approximately 9,000 mutations per genome. Thirty-five out of the 52 flax *cad* mutant families containing missense or codon stop mutations showed the typical orange-brown xylem phenotype observed in *CAD* down-regulated/mutant plants in other species.

**Conclusions:**

We have developed a flax mutant population that can be used as an efficient forward and reverse genetics tool. The collection has an extremely high mutation rate that enables the detection of large numbers of independant mutant families by screening a comparatively low number of M2 families. The population will prove to be a valuable resource for both fundamental research and the identification of agronomically-important genes for crop improvement in flax.

## Background

Flax (*Linum usitatissimum* L.) is an economically important oil and fiber crop that has been domesticated and grown by mankind for thousands of years. Oil extracted from flax seeds (linseed) is a considerable source of the omega-3 fatty acid, α-linolenic acid (ALA) and seeds also contain biologically active lignans with beneficial effects on human health [1]. Flax phloem fibers have cell walls rich in cellulose and are used for textiles (linen) and for reinforcing composite polymers as an environmentally-friendly substitute for glass fibers [2]. Flax is also used as a biological model to study the molecular mechanisms involved in the formation of hypolignified secondary cell walls characteristic of different fiber species (e.g. flax, hemp, jute, kenaf etc.) [3-6].

Recently a number of different flax resources and approaches including high-density microarray platforms, physical and genetic maps, molecular markers, metabolomics and proteomics [6-12] have been developed. The recent sequencing of the genome [13] has also opened the way for flax genomics leading to rapid advances in the structural identification of genes and gene families [14,15]. However, while all of these approaches allow the identification of large numbers of genes potentially involved in a wide variety of different biological processes, the confirmation of their biological role(s) requires functional characterization. Flax can be genetically engineered and a limited number of genes have been up-/down-regulated in this species thereby providing important functional information on the role of these genes [5,16,17]. In order to accelerate functional characterization of genes potentially associated with different agronomical traits for crop improvement we have developed a chemically mutagenized (EMS) flax population and TILLinG (Targeting Induced Local Lesions IN Genomes) platform.

TILLinG (Targeting Induced Local Lesions IN Genomes) is a high-throughput reverse genetic method used to obtain an allelic series of a targeted mutated gene in a mutagenized population [18,19]. Chemical mutagenesis is complementary to other approaches such as T-DNA insertion or radiation and has been applied to a wide range of different plant species [20-24]. Currently, the most usual detection method depends on the use of the specific mis-match endonuclease ENDO1 to detect chemically induced SNPs. Nevertheless, high throughput sequencing by NGS technologies coupled with variant detection algorithms is also starting to be used to detect such mutations [25,26]. The development of such an approach in flax is timely since although this species can be transformed by *Agrobacterium*, the process is time consuming and relatively inefficient [27]. Ethane Methyl-Sulphonate (EMS) has been previously used as a chemical mutagen to introduce genetic variability into flax, but has not yet been used for reverse genetics [28].

In this paper, we present the development and characterization of a flax EMS mutagenized population. Visual phenotyping and the successful identification of a large number of *cad* and *c3h* lignin gene mutants validated the use of our population as a valuable forward and reverse genetics tool. The use of this population in subsequent studies will greatly facilitate the functional characterization of different targeted genes in this economically important species.

## Results

### Production and phenotyping of the EMS Flax mutant population

Preliminary tests and kill curve analyses (Additional file 1) were performed on flax (*Linum usitatissimum* L. cv Diane) seeds to determine appropriate EMS concentrations as previously described [29-32]. Based on these results 3 different EMS percentages (0.3%, 0.6% and 0.75%) were used to mutagenize 10,000 flax seeds. These mutagenized seeds were sown and gave rise to 5,000 M1 plants. M2 seeds were collected from M1 plants and all the seeds produced by an individual M1 plant were pooled to constitute the corresponding M2 family. After seed collection from M1 plants, 4,894 M2 different seed families were obtained and used as the basis for the flax TILLinG resource.

Five (0.3, 0.6% EMS) and 3 (0.75% EMS) seeds from each M2 family were then sown and grown under greenhouse conditions to produce M2 plants for phenotyping and DNA extraction. Altogether, a total of 10,839 M2 plants distributed in 4,033 families (0.3% - 1,700 families, 0.6% - 1,409 families, 0.75% - 924 families, Table 1, Additional file 2) was obtained. Seeds from 861 M2 families (17.6%) did not germinate. All M2 plants were individually phenotyped on the basis of 6 main categories and 13 sub-categories (Table 2) at 2 months after germination. Mutant families were compared to WT plants grown under identical conditions in the same greenhouse.

**Table 1:** Constitution of the M2 mutant population and percentage sterility for each EMS concentration

**% EMS**	**Number of M2 families**	**Percentage sterility**
0.3	1,700	6.3
0.6	1,409	13.5
0.75	924	18.4

**Table 2:** Criteria of Phenotyping categories used to describe the Flax M2 mutant population

**Category**	**Sub-category**
Cotyledon:	Number
	Shape
Hypocotyl:	Size
Stem:	Size
	Diameter
	Colour
Leaf:	Shape
	Colour
Architecture:	Branching type
	Inter-nodes
Flowering/Fruit:	Inflorescence
	Petal colour
	Reproductive organs

**Figure 1:**
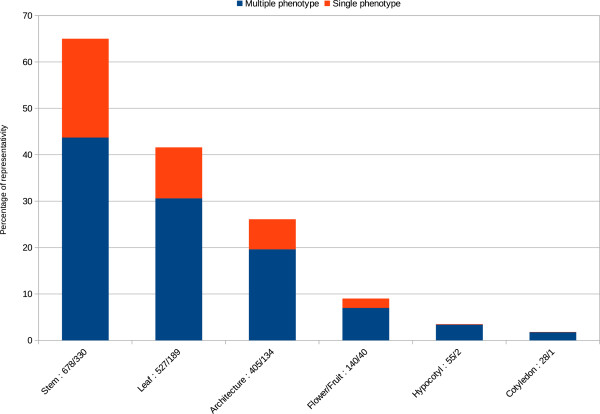
**Flax M2 mutant distribution in different visual phenotype categories.** Blue column = mutiple phenotype, orange column = single phenotype, figures refer to number of M2 families showing multiple/single phenotypes for a given category.

**Figure 2:**
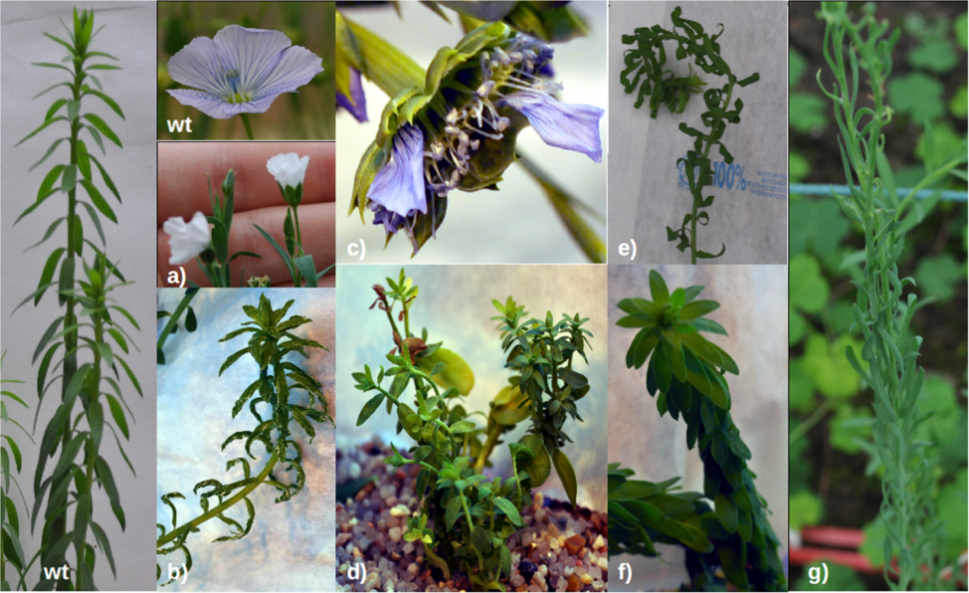
**Examples of mutant flax phenotypes. a)** Flowering mutant with altered petal colour; **b)** albino mutant; **c)** flowering mutant with additional reproductive and non-reproductive organs; **d)** mutant with branching and stem size changes; **e)** mutant with altered leaf morphology (rolled leaves); **f)** mutant with reduced internodes; **g)** mutant with altered leaf morphology (elongated spoonbill shape).

Of the 4,033 M2 families, 1,552 (38.5%) showed a visual phenotype (Figures 1 and 2). The most represented phenotypes were observed in stems (size and diameter – 65%), leaves (shape and colour – 46.1%) and plant architecture (branching internode size – 26.1%), followed by flower-related phenotypes (late flowering and/or fruit formation, colour, morphology – 9%), hypocotyl size (3.5%) and cotyledons (shape and number – 1.8%). A majority (55.2%) of the families show multiple phenotype modifications (Figure 1). Approximately 8.6% (937) M2 plants were sterile and analyses indicated that sterility varied depending upon EMS concentration ranging from 6.31% (0.3% EMS) to 18.36% (0,75% EMS) (Table 1).

Data on flax mutant phenotypes were introduced into the UTILLdb database and can be consulted at http://urgv.evry.inra.fr/UTILLdb.

### Flax lignin gene TILLinG

For reverse genetics, leaves of individual M2 plants were collected and pooled by families for DNA extraction. M3 seeds were collected for long-term storage and production of further material. To estimate mutation density and validate the flax population for future reverse genetics in this species, two genes were TILLed. Since flax is cultivated for both its seeds and its bast fibers and cell wall lignin content is an important factor in bast fiber quality, we decided to TILL two lignin genes (*coumarate-3-hydroxylase, C3H* and *cinnamyl alcohol dehydrogenase, CAD*). C3H acts early in the monolignol biosynthetic pathway and plays a key role in the production of G and S lignin monomers whereas CAD catalyses the final step in the production of the lignin monomers (monolignols) [33].

**Figure 3:**
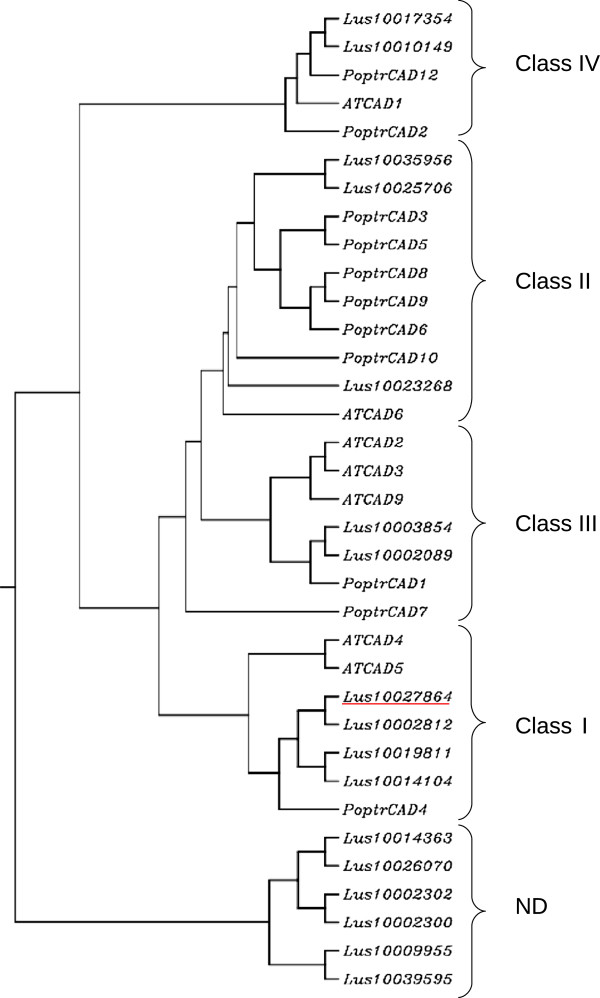
**Rooted Phylogenetic tree (clustalW program with default parameters) of *Arabidopsis thaliana *, Poplar and *Linum usitatissimum *L. CAD proteins.** The flax CAD protein selected for mutation discovery is underlined in red.

Based on currently available sequence data (http://www.phytozome.net/) provided by whole genome sequencing, three *C3H* and seventeen *CAD* genes can be identified in the flax genome. For *C3H* we decided to screen for mutations in [Phytozome : Lus10033524] the flax ortholog of the *Arabidopsis thaliana* CYP98A3 [Tair : At2G40890.1] involved in lignin and flavonoid biosynthesis [34]. In order to identify the flax *CAD* gene most likely involved in lignification, we established a phylogenetic tree containing CAD proteins from *Arabidopsis thaliana*, *Populus trichocarpa* and *Linum usitatissimum* (Figure 3). CAD proteins can be divided into 5 classes [35] with functional proof for the involvement of class 1 proteins in lignification. Four *LuCAD* genes were present in this class and we therefore decided to screen for mutations in the flax gene [phytozome : Lus10027864] showing the highest similarity with AtCAD4 [Tair : AT3G19450.1] and AtCAD5 [Tair : AT4G34230.1].

The CODDLE program (Codons Optimized to Discover Deleterious Lesions) combined with the PRIMER 3 tool were used to define the region within our two genes, with the most probability of a deleterious G/C to A/T transition and to design PCR primers compatible with the ENDO1 approach. A region of 1,077 Nt (1170–2247) was identified in the *C3H* [Phytozome : Lus10033524] gene. For the *CAD* gene [Phytozome : Lus10027864], CODDLE targeted a region between position 38 and 1336 in the genomic sequence, corresponding to the dehydrogenase alcohol domain. However, given sequence redundancy between different flax *CAD* genes in this region we chose to target the region between positions 828 and 1791. This region is composed of a nucleotide binding domain involved in cofactor binding and a catalytic domain involved in substrate binding [36].

A total of 150 *CAD* and *C3H* mutants was obtained by screening for mutations with the mismatch-specific endonuclease ENDO1 as previously described [37]. Sixty-three, 49 and 39 mutations were detected in the 0.3%, 0.6% and 0.75% EMS populations, respectively. One *cad* family (0,6%) contained 2 mutations. Mutation densities varied between 1/49 kb and 1/30 kb depending on the EMS dose giving rise to an average value of 1 mutation per 41 kb.

**Figure 4:**
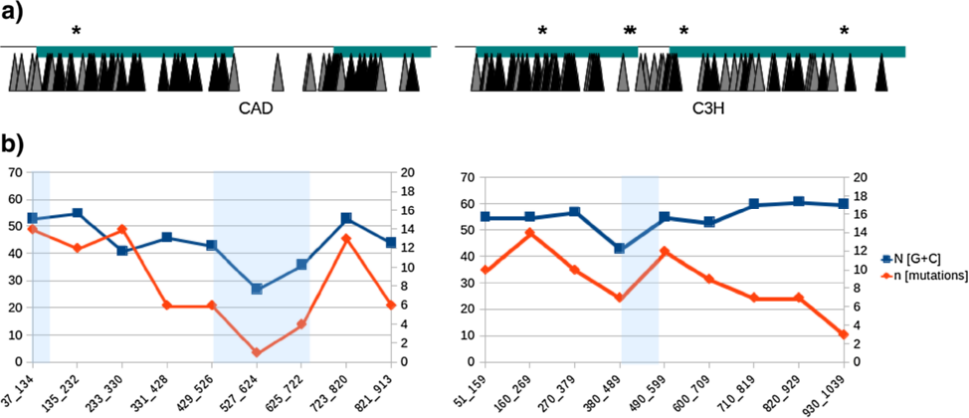
**Distribution of mutations within *CAD *and *C3H *amplicons. a)** Schematic representation of mutation distribution along the amplicons. Introns and exons are represented by black lines and blue boxes, respectively. Missense, silent and truncation mutation are represented by black triangles, grey triangles and asterisks, respectively. **b)** Relationship between number of mutations and G + C content. Amplicons were divided into 100 bp fragments and the numbers of [G + C] and mutations determined. Introns are represented by light blue shading.

Almost all mutations were G/C to A/T transitions, as expected for EMS mutagenesis [38] and only one mutation involved an A/T to G/C transition most likely due to low frequency EMS-mediated conversion of adenine to 3-methyladenosine and subsequent paring with cytosine as already observed in a previous study [39]. For mutations that occurred in exons, 66.7% were missense, 27.6% silent and 5.7% truncation mutations (Table 3). The mutation saturation (at the protein level) was calculated as 22.15% and 15.35% for the *CAD* and *C3H* amplicons, respectively. Mutations were regularly distributed along both amplicons, but showed a decrease in introns corresponding to a decrease in G and C nucleotides in these regions (Figure 4).

**Table 3:** **Characteristics of mutations in *CAD *and *C3H *amplicons**

**Target**	**Amplicon size**	**Identified mutations**	**Mutation frequency**	**Exon size**	**% of GC in exons**	**Identified mutations in exon**	**Type of mutation (exon)**			**% Mutation saturation**
							**Silent**	**Missense**	**Truncation**	
CAD	869	76	1/40 Kb	620	50.0	67	15	51	1	22.5
							(22.4%)	(76.1%)	(1.5%)	
C3H	981	79	1/44 Kb	870	52.53	74	24	43	7	15.35
							(32.4%)	(58.1%)	(9.5%)	

Silent mutations do not modify amino acids, missense mutations induce amino-acid modifications, truncation mutations induce a premature STOP codon in the reading frame. Percentage mutation saturation is calculated at the protein level as the number of amino acids modified compared to the total number of amino acids that can be potentially modified by EMS action.

### EMS action on G/C base pairs is influenced by the local environment

**Figure 5:**
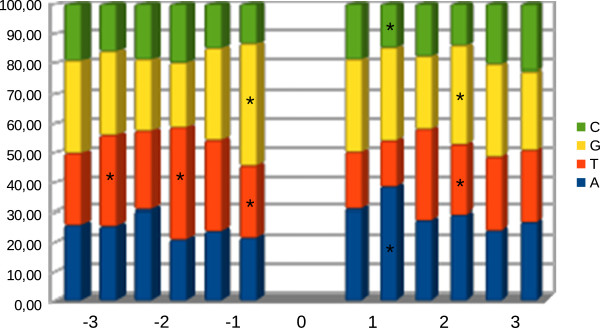
**Expected and observed base frequencies around Gs. Expected (left column) and observed (right column) frequencies of C, G, T, A bases around all Gs or all mutated Gs.** A divergence of ≥15% between expected and observed frequencies is indicated by an asterisk.

In order to investigate the influence of local environment on EMS directed G/C to A/T transition we analyzed nucleotide composition flanking the mutated G bases [40,41] (Figure 5). In the -1 position, G was more frequent (1.3x) and T was less frequent (0.8x) and in the +1 position, A was more frequent (1.2x) and C was less frequent (0.8x) than expected in agreement with previous results [40,41]. We also observed a bias among the observed and expected triplets composed of -1; 0 and +1 nucleotides (Table 4). The four most overrepresented mutated triplets were GGG, CGT, AGA and GGA, whereas the four most underrepresented triplets were AGT, CGC, TGT and CGG, corresponding to a higher frequency of purine bases in the overrepresented triplets and an increase in pyrimidine bases in the underrepresented triplets.

**Table 4:** Frequency of triplet motif centred on all Gs and on mutated Gs. Ratios were obtained by comparing the frequency of a particular triplet centred on the mutated G to the frequency of the same triplet centred on any available G in both amplicons

**Triplet**	**Frequencies of triplet centered on all Gs**	**Frequencies of triplet centered on mutated Gs**	**Ratio**
GGG	7.69	14.29	1.86
CGT	2.83	5.19	1.83
AGA	6.68	10.39	1.56
GGA	10.32	14.94	1.45
TGA	8.50	8.44	0.99
CGA	5.26	5.19	0.99
GGT	6.68	6.49	0.97
TGC	5.47	5.19	0.95
GGC	6.07	5.19	0.86
AGC	4.86	3.90	0.80
AGG	8.30	6.49	0.78
TGG	10.53	7.14	0.68
CGG	4.45	2.60	0.58
TGT	5.87	3.25	0.55
CGC	2.63	0.65	0.25
AGT	3.85	0.65	0.17

### From the genotype to the phenotype

Having identified a large number of flax *CAD* and *C3H* mutants we then wished to know whether the mutations were associated with phenotypic modifications commonly observed in down-regulated plants or natural mutants in other species. Such data would also largely contribute to validating the interest of our mutant population for functional genomics in flax. For this we decided to focus on the *CAD* mutants since both natural *CAD* mutants and down-regulated *CAD* plants in other species show a characteristic and easily observable red-brown coloration of the wood known as the 'brown midrib’ phenotype. This coloration is mainly due to the accumulation of higher amounts of cinnamaldehydes into the lignin polymer rather than the more usual cinnamyl alcohols [42-48]. In contrast *C3H* down-regulation in other species is associated with modifications in lignin structure but no visible phenotype [49]. Observation of stem cross-sections obtained from the 52 M2 mutant CAD families (108 plants) with a missense or stop codon mutation allowed us to identify 62 plants (belonging to 35 families) with a brown midrib phenotype (phenotype frequency = 0.57). A comparable screening of a randomized subset of 33 non-CAD mutant families (94 plants) identified 6 plants with a weak brown midrib phenotype (phenotype frequency = 0.06). These data strongly support the idea that the observed brown midrib phenotype results from a mutation within the targeted flax *CAD* gene.

**Figure 6:**
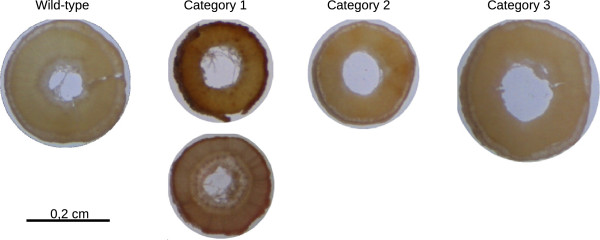
**Brown-midrib phenotype of flax *cad *mutants.** Examples of brown midrib phenotypes observed in stems of *cad* mutant lines. Mutants were grouped into 4 classes according to phenotype intensity (1 = strong, 2 = medium, 3 = weak, 0 = absent). WT = wild-type flax stem. Bar = 2 mm.

Individuals were then classed into 3 categories depending upon the intensity of the orange-brown coloration of the xylem tissues (Figure 6). The first group, corresponding to the most marked phenotype contained 11 individuals belonging to 9 mutant families (8 missense mutations and one codon stop mutation). Characteristics of these mutants are detailed in Table 5. The potential effect of the different missense mutations was then evaluated by using the SIFT software (Sorting Intolerant From Tolerant; http://sift.jcvi.org/) that uses PSI-BLAST alignments, and the PARSESNP software (Project Aligned Related Sequences and Evaluate SNPs; http://www.proweb.org/parsesnp/) to provide a position-specific scoring matrix based on alignment blocks. PSSM scores were obtained by PARESSNP for 5 of the 8 missense mutations (Table 5). No scores were obtained for the G176R and P280L mutations, most probably because of a lack of alignment blocks. In addition to the codon stop mutation, 4 of the six PSSM scores are sufficiently high as to suggest that CAD protein activity could be negatively affected in the corresponding mutants. For the other *CAD* mutants (categories 0, 2 and 3), 33% of the predicted score (PSSM and Sift) were in agreement with the observed phenotype (Additional file 3).

**Table 5:** PSSM/Sift scores for CAD mutant lines showing category 1 brown midrib phenotype

**[EMS]**	**Family**	No. Indiv 1/total*	**Mutation (Nt)**	**Mutation (AA)**	**PSSM difference**	**Sift score**
0,3	645	3/3	G1734A	E351K	**11.0**	0.09
	759	1/3	G1218A	V252I	7.3	0.11
	823	1/5	G1026A	G188R	**30.4**	**0.00**
	1021	1/4	G990A	G176R	_	**0.00**
	1411	1/3	G936A	A158T	**11.0**	0.09
	1564	1/1	C1303T	P280L	_	**0.00**
0.75	814	1/1	C999T	Q179Stop	**damaging**	**damaging**
	877	1/3	C1079T	H205Q	8.0	0.01
	884	1/2	G1137A	G225R	**12.8**	0.09

PSSM scores and Sift scores were obtained with PARSESNP and SIFT programs, respectively. Scores in bold text are predicted to be damaging for protein activity while scores in normal text are predicted to be without effect. Mutations with no scores result from the lack of alignment block for the given position. *Number individual plants showing category 1 phenotype/total number plants phenotyped per family.

## Discussion

**Figure 7:**
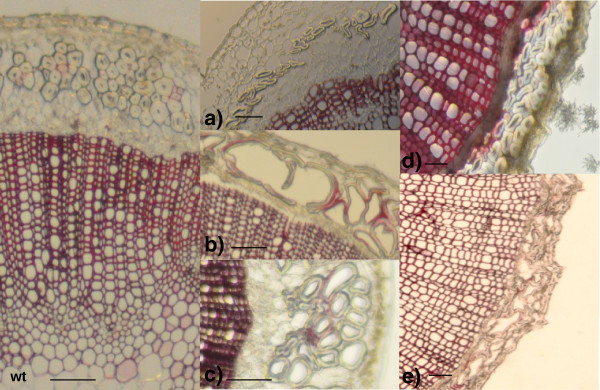
**Examples of flax mutants showing morphological modifications in bast fibers. a)** mutant with low fiber number and fibers with thin secondary cell walls; **b)** mutant with disorganised fiber bundles containing lacuna and thin-walled fibers with wide lumens; **c)** mutant with fibers containing wide lumens; **d)** mutant with flattened bast fibers; **e)** mutant with disorganised fiber bundles containing wide lumens and thin secondary cell walls.Freehand cross-sections of mature flax stems were stained with Phloroglucinol-HCl and lignified cell walls appear coloured in red. Bar = 0,1 mm.

**Figure 8:**
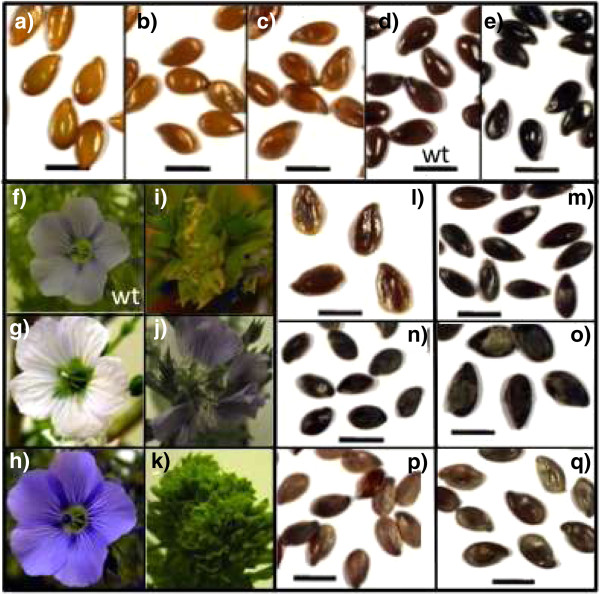
**Examples of seed and flower phenotypes observed in the mutant population.** 1-Seeds of mutants showing different colours from deep yellow **(a)** to deep black **(e)** and in between colour variation **(b**, **c)** including wild type **(d)** (Bar = 0.5 cm). 2-Flowers of mutants showing different colours from white **(g)** to parme variation **(f**, **h)**. 3-Flowers of mutants with abnormal shape **(i**, **j**, **k)**. 4-Seeds of mutants showing variation in shape **(l**, **m)** (Bar = 0.5 cm). 5-Seed size variation between 2 specific mutants **(n**, **o)** (Bar = 0.5 cm). 6-Seeds of mutants more or less filled **(p**, **q)** (Bar = 0.5 cm).

Flax is an ancient crop that has long been cultivated for its fibers and seeds. Current breeding programs aim at improving yield and quality in both fibers and seeds, as well as increasing resistance to different pathogens. The flax genome has recently been sequenced [13], physical and genetic maps have been developed and SNP markers identified [10]. In addition, flax specific microarrays, proteomics and metabolomics [6,7,12,50,51] have also been used to increase our knowledge of flax biology. In this paper we report the development of a flax TILLinG platform for forward and reverse genetics in this economically important species. 4,033 independent mutant families were phenotyped and the results organised in the UTILLdb database (http://urgv.evry.inra.fr/UTILLdb). UTILLdb is an open phenotypic and genomic mutant database containing information on mutant populations of pea [37], Brachypodium [52], tomato [53] and flax (this paper). The integration of our data into UTILLdb will enable flax breeders and scientists working on the flax model to search the database for particular mutant phenotypes. For example, detailed characterization of mutants showing altered stem/fiber morphology (Figures 2 and 7), and/or flower/seed modifications (Figures 2 and 8) will prove particularly interesting for scientists and breeders interested in fiber formation and seed oil/lignan biosynthesis. The database will be updated with information on mutations in specific genes that scientists will be able to access by sequence homology and/or keyword queries.

In order to validate the mutant collection as an efficient reverse genetics tool we extracted DNA from 3,515 lines and successfully TILLed 2 genes involved in lignin biosynthesis using the ENDO1 enzyme as previously described [37,54]. Our results indicated that our population had an average mutation rate of 1/41 Kb. This is a high value as compared to mutation rates observed in other EMS-generated mutant populations (Table 6) and is similar to values observed for *Triticum aestivum* L.; *Brassica napus* L.; *Avena sativa* and *Triticum durum* L*.* However, all these populations are polyploid allowing them to tolerate loss-of-function mutations [55]. The highest mutation rates previously obtained in diploid species are those observed in *Triticum monococcum* (1/92 Kb) and *Arabidopsis thaliana* (1/89 Kb), approximately half that observed in our flax population.

**Table 6:** Mutation frequencies and ploidy levels in different published EMS plant populations

**Model**	**Ploidy level**	**EMS concentration (%)**	**Frequency of mutation**	**Source**
*Linum usitatissimum* L.	2x	-0.3, 0.6, 0.75%	-1/41 kb	
*Triticum monococcum*	2x	-0.24%	-1/92 kb	[32]
*Triticum aestivum* L.	6x	-0.8%	-1/47 kb	[31]
		-0.6, 0.9%	N.A	[56]
		-0.9%	-1/38 Kb	[57]
		-0.5, 0.6, 0.7%	-1/25 Kb	[58]
		-0.75, 1%	-1/24 Kb	[59]
*Triticum durum*	4x	-0.7%	-1/51 Kb	[57]
		-0.75%	-1/40 Kb	[59]
*Brassica napus* L.	4x	-0.5, 0.8, 1, 1.2%	-from 1/12 to 1/22 kb	[60]
		-1%	-from 1/27 to 1/60 kb	
		-0.3%	1/130.8 kb	[61]
		-0.6%	1/41.5 kb	
*Brassica rapa*	2x	-0.3, 0.4%	1/60 Kb	[62]
*Brassica oleracea*	2x	-0.4%	-1/447 Kb	[63]
*Solanum lycopersicum*	2x	-0.5%	-1/1 710 kb	[30]
		- 1.0%	-1/737 kb	
		-0.7%	-1/574 Kb	[53]
		- 1%	-1/322 Kb	
		-1%	-1/737 Kb	[64]
*Cucumis melo* L.	2x	-1%	-1/1500 Kb	[65]
		-1.5, 2%	-1/573 Kb	[66]
*Heliantus annus* L.	2x	-0.7%	-1/475 Kb	[67]
*Arachis hypogaea*	4x	-0.4, 1.2%	-1/967 Kb	[68]
*Arabidopsis thaliana*	2x	-0.2%	-1/415 Kb	[69]
		-Between 0.25 and 0.5%	-1/89 Kb	[55]
		-0.25, 0.5%	-1/300 Kb	[40]
		-Between 0.25 and 0.56 %	-1/180 Kb	[54]
*Avena sativa*	6x	-0.9%	-1/30 kb	[70]
*Glycine max* L. Merr.	2x	-0.2, 0.15%	-1/485 Kb	[41]
*Lotus japonicus*	2x	N.A	-1/502 Kb	[71]
*Hordeum vulgare* L.	2x	-Between 0.25 and 0.75 %	-1/500 Kb	[72]
*Medicago trunculata*	2x	-0.15%	-1/400 Kb	[73]
*Sorghum bicolor*	2x	-Between 0.1 and 0.3%	-1/526 kb	[74]
*Pisum sativum*	2x	-0.25%	-1/200 kb	[37]
		-0.05%	-1/669 Kb	[25]
*Oriza sativa*	2x	-1.5%	-1/294 Kb	[75]
		-0.8, 1%	-1/2 000 Kb	[76]
		-1.6%	-1/1 000 Kb	
*Hordeum vulgare* L.	2x	-0.25, 0.4	-1/1 000 Kb	[77]

With a genome size of approximately 370 Mb [13] we can estimate that there will be an average of approximately 9,000 mutations per genome. Despite this high value, the majority (81.6 – 93.7%) of the M2 population were viable and produced seeds suggesting that flax plants can support a high mutation level [55]. Although the high number of mutations per genome might be considered as a disadvantage since more back-crosses will be necessary to reduce total mutation number and identify genes potentially associated with a particular phenotype via a positional cloning approach, it also presents a number of advantages. Firstly, it considerably reduces the number of families that need to be screened to identify a mutant. For example, calculations show that only 56 families have to be screened to identify a missense mutation in a 1 Kb exon target and only 650 families need to be screened to identify a codon stop mutation, thereby reducing the overall time and costs spent on mutant identification. Secondly, the high mutation rate allows the identification of a large number of independent mutant families for a given gene in a reverse genetics strategy. For example, screening of our total population allowed us to identify 67 *cad* mutants (exons only) and 74 *c3h* mutants (exons only). Subsequent characterization and identification of similar phenotypic modifications in the different lines provides strong evidence for a link between gene mutation and the observed phenotype. This was clearly demonstrated in the observation of our *cad* mutants where 35 (71%) of the 52 families showing a missense or codon stop mutation in the *CAD* gene showed the characteristic orange-brown coloration of xylem tissue previously observed in other *CAD* down-regulated or mutant plants [42-48]. Although a previous report of CAD down-regulation in flax RNAi plants was associated with reduced lignin, somewhat surprisingly the authors did not report the presence/absence of a brown midrib phenotype [16]. This might be related to the relatively high (60–80%) residual activity in these plants.

Preliminary analyses of our M3 *cad* mutants (data not shown) indicate that the mutation is heritable, segregates and can be correlated with brown/orange coloured xylem further establishing the link between mutation and phenotype in the flax population. Wet chemistry and spectroscopy will allow confirmation of structural modifications in cell wall lignin in these mutants. Similar techniques will also be used to investigate potential changes to the lignin polymer in flax *c3h* mutants. Down-regulation of this gene in other species is associated with an increase in lignin condensation and reduction in lignin G and S units [49,78] and it will be particularly interesting to assess the effects in flax lignin that is already highly condensed and contains low amounts of S lignin [3].

## Conclusions

In conclusion, the generated flax EMS population represents an important biological resource for both forward and reverse genetics in this species. A large number of mutants showing biologically-interesting phenotypes has been identified and genes have been successfully TILLed using ENDO1. Further targets can be identified from the literature, as well as on the basis of recent transcriptomic studies in flax that have identified different genes potentially involved in various biological processes [6,7,9]. The use of the flax EMS population to identify mutants will greatly accelerate functional characterization of agronomically-interesting genes in this crop species. In order to accelerate mutant identification in our flax EMS population, we are currently developing an approach based on high throughput sequencing using NGS.

## Methods

### Mutagenesis and plant growth conditions

Ten batches of one hundred seeds (*Linum usitatissimum L.* cv Diane) were treated with 8 different EMS concentrations (0.25, 0.3, 0.5, 0.6, 0.75, 0.8, 1.0 and 2.0%). Two exposure times (5 and 8 h) were tested for two batches of different EMS concentration (5 h: 0.3, 0.6, 0.8, 1.0, 2.0% EMS; 8 h: 0.25, 0.5, 0.75, 1.0, 2.0% EMS). Untreated seeds were used as reference. All seeds were washed with tap water (3 × 5 mins, 1 × 30 mins) before transfer to wet Whatman paper in Petri dishes and incubation in a growth chamber at 20°C and 8 h photoperiod. Based on percentage germination, three treatments (0.3% EMS/5 h, 0.6% EMS/5 h and 0.75% EMS/8 h) were selected representing different balances between germination and mutation rate. Mutagenized seeds (M1) were sown in the field for M2 seed production. Collected M2 seeds were sown under greenhouse conditions together with WT seeds for phenotyping and DNA extraction. M2 and M3 seeds are stored at 4°C under low humidity conditions and constitute the EMS flax mutant collection.

### Forward genetic screen

Two months after germination, M2 families were scored for phenotypes distinct from the wild type. For each family, the most affected individual was phenotyped in detail and photographed. For identification of the brown midrib phenotype, freehand sections of individual stems were made from *cad* mutant family plants and compared to control sections under a stereo microscope. The presence of a brown-orange coloration in xylem tissue was considered indicative of the brown midrib phenotype and was noted from 1 to 3 depending upon the intensity of the coloration with 1 being the most intense. The category indicated for a family is that of the most intense phenotype observed in any single individual belonging to that family.

### Genomic DNA extraction and pooling

Leaf material was collected from individual M2 plants and pooled by family before being dried overnight at 65°C in a ventilated oven. Total DNA was extracted by using either the Dneasy Plant 96 Qiagen Kit (Qiagen, Hilden, Germany) or according to the protocol described by Carrier et al. [79]. DNA quality for each extraction was monitored by electrophoresis on 0.8% agarose gels. The DNA of 3,515 M2 family was quantified with Picogreen (InVitrogen) using a M1000 microplate reader (TECAN-Switzerland), normalized to 1 ng.μL^-1^ and arrayed in a total of five 96 wells plates by an 8-fold pooling strategy (Additional file 4) using a GENESYS 150 workstation (TECAN-Switzerland).

### PCR amplification and mutation detection

PCR amplification was based on nested-PCR and universal primers [80]. The first PCR amplification was performed with 1 ng of pooled genomic DNA and target-specific primers (Table 7) in a 25 μl volume. One microliter of the first PCR reaction was then used as a template for the second PCR using two set of primers: target-specific primers carrying universal M13 tail and M13 universal primers labelled at the 5′ end with infra-red dyes IRD700 and IRD800 (LI-COR, Lincoln, NE, USA). This PCR amplification was performed using 0.1 μM of each primer in a 25 μL volume and with the following two step program: 94°C for 3 minutes; 10 cycles at 94°C for 20 s, primer-specific annealing temperature for 30 s and 72°C for 1 min; and 25 cycles at 94°C for 20 s, 50°C for 30 s and 72°C for 1 min; then a final extension at 72°C for 5 mins. PCR amplifications were verified on an agarose gel. Mutations were detected using a LI-COR 4300 DNA analyzer as previously described [66]. Individual mutations were confirmed and characterized by sequencing of DNA from individual M2 families.

**Table 7:** Primers used in TILLinG experiments

**Primer**	**5′-3′ Primer sequence**
CAD external Forward primer	5′-ACAGTTTGACCTGATGGAGCTCGAT-3′
CAD external Reverse primer	5′-GAAAACAAGTCAAATCGGACATAGG-3′
C3H external Forward primer	5′-ATATTTACCAACCGGACTAACCTTG-3′
C3H external Reverse primer	5′-AGTACAACACAATTCCAACTCTTCG-3′
CAD internal Forward primer with M13 tail	5′-**CACGACGTTGTAAAACGAC**TTTCGGTCCATCATCG-3′
CAD internal Reverse primer with M13 tail	5′-**GATAACAATTTCACACAGG**TATGGGTCTTCTCTTC-3′
C3H internal Forward primer with M13 tail	5′-**CACGACGTTGTAAAACGAC**ACCACACTGAATTCGG-3′
C3H internal Reverse primer with M13 tail	5′-**GATAACAATTTCACACAGG**CATGTAAGTCACCAGT-3′
M13 Forward primer	5′-CACGACGTTGTAAAACGAC-3′
M13 Reverse primer	5′-GGATAACAATTTCACACAGG-3′

The M13 tail sequence is indicated in bold text.

### Bio-informatic sequence analyses

Phylogenetic analyses of CAD proteins was performed using the clustalW program with default parameters (http://www.clustal.org/clustal2/). The choice of gene regions to be TILLed and amplification primers were designed respectively using the CODDLE tool (*Codons Optimized to Discover Deleterious Lesions;*http://www.proweb.org/coddle/*)*, primer 3 and OligoCalc system (http://www.basic.northwestern.edu/biotools/oligocalc.html). Potential effects of missense mutations were evaluated using the SIFT software (Sorting Intolerant From Tolerant; http://sift.jcvi.org/) and the PARSESNP software (Project Aligned Related Sequences and Evaluate SNPs; http://www.proweb.org/parsesnp/).

### Supporting data

The flax phenotypic data is publically available on the UTILLdb ( http://urgv.evry.inra.fr/UTILLdb).

## Competing interests

The authors declare that they have no competing interests.

## Authors’ contributions

MChant. participated in plant phenotyping and sample collection. He did fiber screening and microscopy, data centralization for the UTILLdb, DNA extraction, all TILLinG experiments and data interpretation. He wrote the manuscript. SG managed the organization of plant phenotyping and sample collection, participated in sample collection, data centralization and DNA extraction. LG participated in plant phenotyping, sample collection, normalization of DNA pools and checked the final manuscript. MD provided scientific expertise and practical help on ENDO1 TILLinG and checked the final manuscript. RT managed greenhouse/field mutant plant production, participated in plant phenotyping and sample collection and checked the final manuscript. J-PT managed greenhouse/field mutant plant production. BT and OvW participated in plant phenotyping, in the obtention of seed collections, of the phenotyping analysis of all seed banks. MChat. Designed the EMS pilot dose experiments, selected the doses and checked the final manuscript. XG represented Laboulet in the PT-Flax consortium. VB organized the phenotypic data set in the UTILLdb web site. AB provided scientific expertise and advice on TILLinG and checked the final manuscript. BC, HD, CT, CP, CP-L, BT, OvW participated in plant growth, phenotyping and sample collection and checked the final manuscript. SH conceived and managed the PT-Flax project, participated in plant phenotyping and sample collection and edited the final manuscript. All authors read and approved the final manuscript.

## Supplementary Material

Additional file 1 **EMS kill curve analyses for flax** (***Linum usitatissimum L.*cv Diane) seeds.** Click here for file

Additional file 2 Number of individual plants phenotyped per M2 family. Click here for file

Additional file 3 **PSSM/Sift scores and phenotypes for CAD mutant lines.** PSSM scores and Sift scores were obtained with PARSESNP and SIFT programs, respectively. Scores in bold text are predicted to be damaging for protein activity while scores in normal text are predicted to be without effect. Mutations with no scores result from the lack of alignment block for the given position. The intensity (0, 1, 2, 3) of the brown-midrib phenotype (orange coloured xylem) in different CAD mutant lines is indicated in the column 'phenotype class’ and corresponds to Figure 5. Class 0 corresponds to wt phenotype. ND = not determined. Numbers in brackets after mutation (first column) indicate the number of families for which a given mutation was independently found. The absence of a number in brackets indicates that the mutation was only found in a single family. Click here for file

Additional file 4 Eight-fold 1D-pooling scheme. Click here for file
