# Supplementary figures and images for: PT-Flax (phenotyping and TILLinG of flax): development of a flax (Linum usitatissimum L.) mutant population and TILLinG platform for forward and reverse genetics
Source: BMC Plant Biol. 2013 Oct 15;13:159. doi: 10.1186/1471-2229-13-159 (PMC3853753; doi:10.1186/1471-2229-13-159)

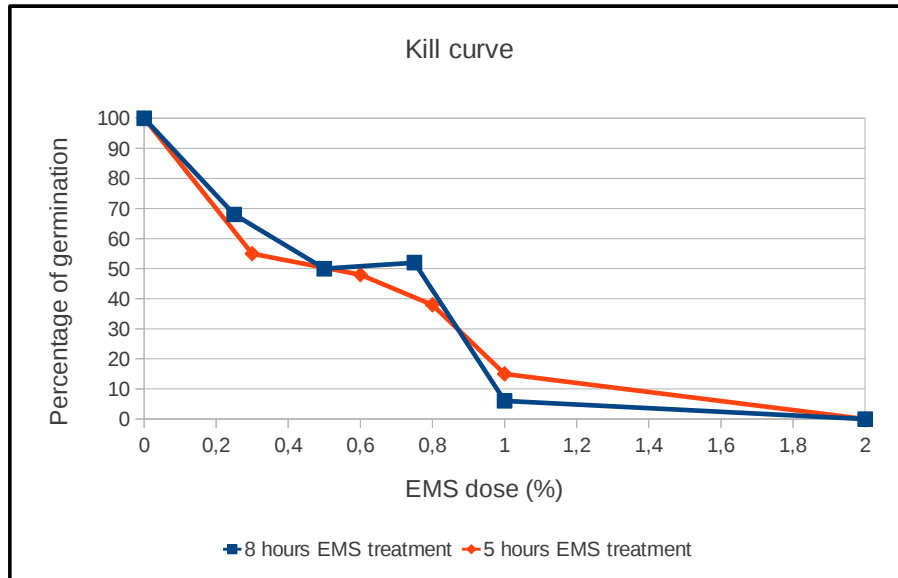

Supplement: Additional file 1 — EMS kill curve analyses for flax (Linum usitatissimum L.cv Diane) seeds. [file 1471-2229-13-159-S1.pdf]

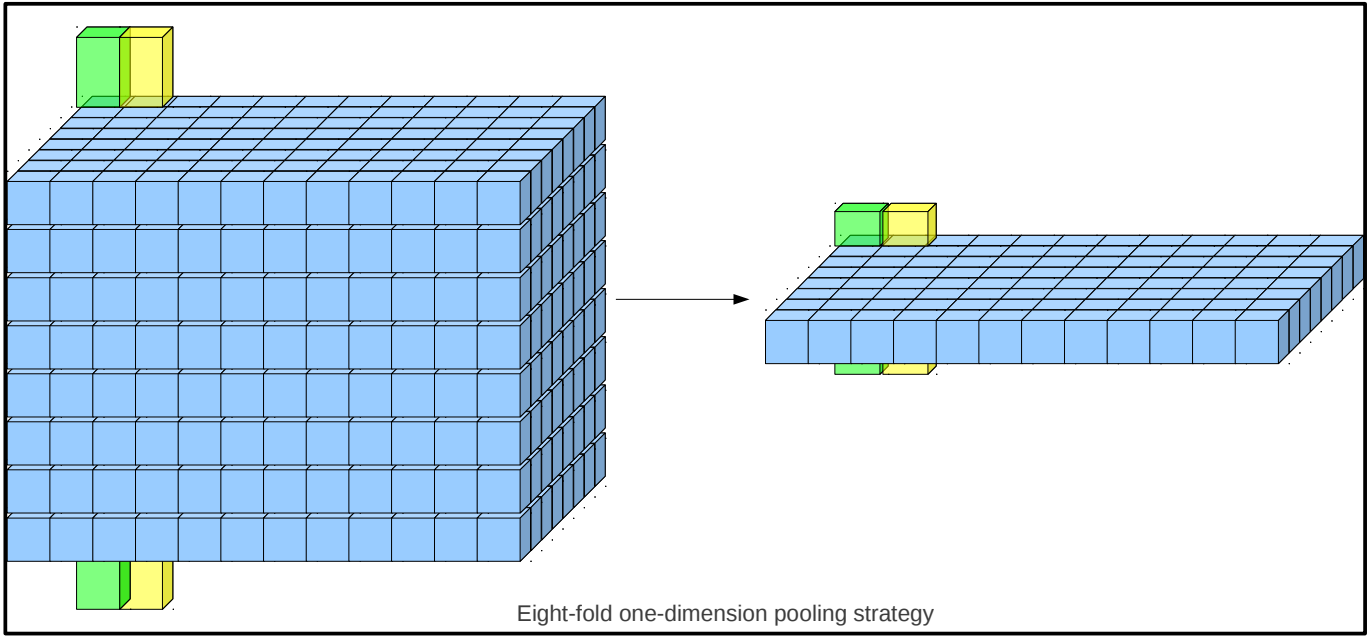

Supplement: Additional file 4 — Eight-fold 1D-pooling scheme. [file 1471-2229-13-159-S4.pdf]
