# Supplementary material for: PT-Flax (phenotyping and TILLinG of flax): development of a flax (Linum usitatissimum L.) mutant population and TILLinG platform for forward and reverse genetics
Source: BMC Plant Biol. 2013 Oct 15;13:159. doi: 10.1186/1471-2229-13-159 (PMC3853753; doi:10.1186/1471-2229-13-159)
Supplement: Additional file 2 — Number of individual plants phenotyped per M2 family. [file 1471-2229-13-159-S2.pdf]

Supplementary data 2 :

EMS : 0.75%	
Family	Number of plants
1	3
2	0
3	0
4	2
5	2
6	2
7	3
8	2
9	2
10	0
11	1
12	3
13	2
14	3
15	2
16	2
17	1
18	1
19	2
20	3
21	3
22	2
23	3
24	3
25	2
26	2
27	2
28	3
29	2
30	1
31	4
32	2
33	1
34	2
35	3
36	2
37	3

EMS : 0.6%	
Family	Number of plants
1	3
2	0
3	2
4	4
5	5
6	6
7	0
8	1
9	3
10	3
11	1
12	3
13	2
14	1
15	1
16	4
17	2
18	3
19	2
20	0
21	3
22	3
23	4
24	4
25	0
26	2
27	4
28	3
29	5
30	4
31	3
32	2
33	0
34	4
35	1
36	3
37	0

EMS : 0.3%	
Family	Number of plants
1	3
2	4
3	1
4	4
5	4
6	4
7	4
8	4
9	3
10	1
11	3
12	3
13	0
14	4
15	3
16	4
17	1
18	3
19	4
20	3
21	5
22	5
23	3
24	4
25	4
26	3
27	3
28	3
29	3
30	5
31	2
32	4
33	3
34	1
35	2
36	4
37	4

38	1
39	4
40	3
41	4
42	3
43	3
44	2
45	2
46	4
47	1
48	3
49	2
50	2
51	1
52	1
53	0
54	2
55	2
56	3
57	3
58	3
59	2
60	3
61	3
62	2
63	2
64	3
65	2
66	2
67	2
68	1
69	3
70	1
71	1
72	1
73	3
74	3
75	2
76	2
77	2
78	2
79	2
80	3

38	2
39	4
40	3
41	2
42	4
43	1
44	0
45	0
46	3
47	4
48	4
49	2
50	3
51	2
52	3
53	3
54	3
55	1
56	0
57	0
58	3
59	1
60	4
61	1
62	3
63	1
64	2
65	2
66	5
67	2
68	1
69	1
70	0
71	3
72	2
73	2
74	2
75	0
76	1
77	2
78	2
79	1
80	2

38	5
39	0
40	2
41	5
42	3
43	4
44	3
45	3
46	2
47	5
48	1
49	5
50	2
51	4
52	0
53	2
54	5
55	2
56	3
57	5
58	3
59	2
60	4
61	5
62	3
63	2
64	3
65	2
66	3
67	3
68	4
69	3
70	4
71	0
72	2
73	0
74	3
75	2
76	3
77	3
78	4
79	3
80	2

81	1
82	2
83	0
84	3
85	1
86	1
87	3
88	3
89	3
90	1
91	3
92	2
93	2
94	1
95	3
96	2
97	0
98	2
99	1
100	2
101	1
102	1
103	2
104	3
105	1
106	1
107	1
108	3
109	2
110	2
111	2
112	3
113	1
114	2
115	2
116	4
117	3
118	2
119	2
120	2
121	2
122	2
123	1

81	0
82	2
83	5
84	1
85	3
86	1
87	3
88	0
89	1
90	2
91	4
92	2
93	0
94	0
95	3
96	3
97	1
98	3
99	3
100	2
101	2
102	2
103	0
104	2
105	4
106	0
107	0
108	0
109	0
110	0
111	0
112	1
113	1
114	0
115	1
116	4
117	2
118	0
119	2
120	3
121	0
122	3
123	0

81	3
82	2
83	4
84	3
85	4
86	2
87	3
88	3
89	0
90	2
91	5
92	3
93	2
94	2
95	4
96	0
97	3
98	4
99	3
100	2
101	3
102	3
103	1
104	3
105	2
106	2
107	4
108	4
109	4
110	1
111	5
112	0
113	0
114	2
115	2
116	3
117	4
118	4
119	3
120	3
121	3
122	1
123	4

124	0
125	0
126	3
127	3
128	0
129	2
130	2
131	2
132	2
133	0
134	1
135	2
136	1
137	3
138	1
139	1
140	2
141	1
142	0
143	3
144	2
145	2
146	3
147	1
148	2
149	3
150	0
151	2
152	2
153	2
154	3
155	0
156	3
157	0
158	3
159	1
160	1
161	1
162	2
163	1
164	0
165	2
166	1

124	4
125	1
126	1
127	3
128	0
129	2
130	2
131	0
132	0
133	3
134	0
135	1
136	0
137	5
138	2
139	1
140	2
141	2
142	3
143	0
144	1
145	3
146	2
147	1
148	1
149	1
150	3
151	3
152	0
153	3
154	2
155	4
156	3
157	1
158	4
159	5
160	0
161	3
162	3
163	0
164	1
165	2
166	1

124	4
125	1
126	3
127	3
128	5
129	3
130	2
131	3
132	0
133	1
134	1
135	1
136	2
137	3
138	2
139	3
140	4
141	3
142	3
143	4
144	3
145	2
146	3
147	5
148	1
149	3
150	4
151	1
152	3
153	2
154	1
155	0
156	5
157	2
158	2
159	2
160	4
161	5
162	3
163	3
164	5
165	5
166	5

167	1
168	0
169	4
170	2
171	1
172	1
173	2
174	0
175	2
176	0
177	2
178	3
179	2
180	2
181	1
182	0
183	2
184	1
185	3
186	2
187	1
188	0
189	2
190	2
191	2
192	2
193	3
194	0
195	2
196	3
197	1
198	2
199	2
200	2
201	1
202	0
203	2
204	2
205	2
206	2
207	0
208	3
209	3

167	4
168	0
169	3
170	4
171	3
172	2
173	0
174	3
175	3
176	3
177	2
178	2
179	4
180	4
181	1
182	1
183	2
184	3
185	0
186	2
187	2
188	1
189	4
190	1
191	4
192	2
193	2
194	4
195	0
196	3
197	4
198	3
199	5
200	0
201	3
202	2
203	3
204	3
205	3
206	3
207	0
208	4
209	3

167	2
168	3
169	3
170	2
171	5
172	2
173	0
174	5
175	3
176	3
177	5
178	0
179	5
180	2
181	5
182	4
183	2
184	5
185	4
186	4
187	4
188	3
189	5
190	4
191	3
192	3
193	5
194	4
195	2
196	3
197	4
198	2
199	3
200	4
201	0
202	2
203	3
204	5
205	2
206	1
207	2
208	4
209	0

210	0
211	1
212	1
213	0
214	1
215	1
216	0
217	0
218	1
219	1
220	3
221	2
222	1
223	1
224	2
225	2
226	0
227	1
228	0
229	1
230	2
231	2
232	2
233	3
234	2
235	1
236	3
237	2
238	2
239	3
240	2
241	3
242	3
243	2
244	1
245	3
246	1
247	2
248	0
249	0
250	3
251	2
252	2

210	3
211	3
212	1
213	0
214	0
215	0
216	1
217	3
218	3
219	2
220	1
221	4
222	2
223	4
224	6
225	0
226	5
227	4
228	1
229	3
230	3
231	0
232	2
233	0
234	4
235	4
236	2
237	3
238	3
239	4
240	3
241	0
242	3
243	4
244	2
245	4
246	3
247	4
248	2
249	0
250	1
251	3
252	3

210	4
211	3
212	1
213	1
214	0
215	5
216	2
217	4
218	3
219	3
220	3
221	4
222	4
223	2
224	4
225	4
226	3
227	1
228	1
229	4
230	2
231	0
232	3
233	2
234	5
235	5
236	4
237	3
238	4
239	3
240	2
241	2
242	3
243	0
244	4
245	4
246	4
247	3
248	4
249	4
250	1
251	2
252	0

253	2
254	3
255	3
256	2
257	3
258	4
259	3
260	0
261	1
262	1
263	0
264	1
265	1
266	2
267	3
268	2
269	1
270	3
271	3
272	0
273	2
274	2
275	0
276	1
277	2
278	2
279	2
280	2
281	1
282	1
283	0
284	3
285	3
286	2
287	3
288	3
289	2
290	4
291	2
292	2
293	2
294	3
295	1

253	3
254	1
255	2
256	3
257	1
258	1
259	4
260	0
261	1
262	1
263	1
264	3
265	2
266	0
267	2
268	4
269	4
270	0
271	1
272	3
273	2
274	3
275	2
276	2
277	3
278	0
279	0
280	0
281	0
282	0
283	3
284	2
285	3
286	1
287	2
288	4
289	1
290	1
291	3
292	0
293	4
294	3
295	4

253	3
254	3
255	3
256	3
257	4
258	0
259	1
260	3
261	4
262	4
263	3
264	0
265	5
266	5
267	3
268	3
269	4
270	3
271	3
272	1
273	0
274	2
275	2
276	3
277	3
278	4
279	3
280	5
281	4
282	2
283	3
284	1
285	4
286	3
287	4
288	2
289	2
290	3
291	3
292	4
293	4
294	4
295	3

296	0
297	3
298	3
299	2
300	4
301	2
302	0
303	2
304	2
305	1
306	0
307	2
308	3
309	3
310	1
311	2
312	4
313	2
314	2
315	1
316	1
317	1
318	0
319	0
320	1
321	1
322	1
323	3
324	3
325	3
326	2
327	1
328	1
329	3
330	2
331	2
332	1
333	1
334	1
335	3
336	1
337	2
338	3

296	3
297	2
298	3
299	3
300	1
301	2
302	1
303	3
304	1
305	0
306	0
307	2
308	2
309	3
310	1
311	4
312	2
313	0
314	1
315	3
316	3
317	3
318	2
319	2
320	3
321	3
322	1
323	4
324	5
325	1
326	0
327	1
328	3
329	2
330	2
331	3
332	4
333	0
334	1
335	1
336	0
337	4
338	0

296	2
297	3
298	4
299	1
300	2
301	0
302	1
303	1
304	2
305	0
306	0
307	5
308	3
309	1
310	5
311	0
312	0
313	0
314	0
315	0
316	1
317	0
318	0
319	0
320	0
321	0
322	0
323	0
324	0
325	0
326	1
327	4
328	3
329	0
330	0
331	0
332	5
333	0
334	0
335	0
336	5
337	0
338	4

339	1
340	2
341	2
342	2
343	1
344	3
345	0
346	3
347	2
348	2
349	3
350	1
351	1
352	2
353	1
354	2
355	2
356	2
357	0
358	3
359	1
360	2
361	2
362	1
363	3
364	1
365	3
366	0
367	0
368	3
369	3
370	0
371	2
372	3
373	2
374	2
375	2
376	2
377	1
378	2
379	1
380	1
381	2

339	1
340	0
341	0
342	3
343	0
344	2
345	3
346	6
347	2
348	3
349	0
350	2
351	1
352	0
353	0
354	4
355	0
356	3
357	2
358	1
359	3
360	3
361	0
362	3
363	2
364	1
365	1
366	3
367	1
368	4
369	0
370	2
371	2
372	3
373	2
374	2
375	0
376	1
377	4
378	1
379	4
380	2
381	2

339	3
340	3
341	0
342	2
343	0
344	5
345	1
346	3
347	3
348	4
349	4
350	4
351	2
352	4
353	5
354	5
355	2
356	0
357	4
358	5
359	0
360	0
361	4
362	3
363	3
364	4
365	4
366	4
367	4
368	2
369	0
370	4
371	1
372	4
373	2
374	0
375	0
376	4
377	0
378	4
379	0
380	3
381	4

382	1
383	3
384	3
385	2
386	3
387	1
388	2
389	3
390	2
391	1
392	1
393	1
394	1
395	4
396	1
397	1
398	2
399	3
400	2
401	2
402	1
403	1
404	0
405	1
406	2
407	3
408	3
409	0
410	2
411	2
412	2
413	3
414	0
415	2
416	2
417	3
418	3
419	1
420	4
421	3
422	1
423	2
424	1

382	1
383	2
384	2
385	2
386	0
387	5
388	3
389	2
390	2
391	5
392	2
393	2
394	5
395	4
396	3
397	4
398	1
399	1
400	5
401	0
402	0
403	4
404	0
405	1
406	3
407	4
408	2
409	4
410	4
411	0
412	2
413	5
414	2
415	2
416	3
417	1
418	4
419	3
420	2
421	0
422	3
423	0
424	2

382	3
383	0
384	1
385	2
386	3
387	1
388	1
389	4
390	0
391	0
392	0
393	0
394	0
395	4
396	0
397	4
398	3
399	4
400	3
401	0
402	4
403	0
404	3
405	3
406	3
407	2
408	4
409	0
410	1
411	4
412	1
413	0
414	0
415	2
416	4
417	2
418	2
419	0
420	2
421	1
422	0
423	0
424	0

425	1
426	2
427	2
428	2
429	2
430	2
431	3
432	2
433	2
434	3
435	1
436	0
437	2
438	2
439	3
440	4
441	2
442	3
443	3
444	2
445	1
446	3
447	1
448	1
449	3
450	1
451	3
452	3
453	2
454	2
455	1
456	0
457	2
458	1
459	3
460	1
461	3
462	4
463	4
464	3
465	3
466	3
467	1

425	3
426	1
427	4
428	1
429	1
430	1
431	1
432	0
433	5
434	5
435	0
436	3
437	0
438	2
439	3
440	3
441	4
442	2
443	2
444	2
445	1
446	2
447	1
448	2
449	2
450	0
451	4
452	4
453	0
454	0
455	0
456	2
457	3
458	2
459	3
460	1
461	1
462	1
463	3
464	3
465	2
466	3
467	3

425	4
426	3
427	4
428	3
429	3
430	0
431	0
432	3
433	0
434	4
435	4
436	2
437	0
438	0
439	5
440	0
441	0
442	0
443	0
444	0
445	4
446	0
447	0
448	4
449	0
450	0
451	0
452	0
453	4
454	0
455	2
456	0
457	0
458	4
459	3
460	4
461	2
462	1
463	1
464	4
465	1
466	0
467	4

468	1
469	1
470	1
471	3
472	1
473	1
474	3
475	1
476	2
477	4
478	4
479	3
480	2
481	1
482	2
483	3
484	3
485	1
486	1
487	1
488	2
489	3
490	3
491	2
492	3
493	3
494	2
495	3
496	3
497	2
498	2
499	3
500	3
501	3
502	4
503	4
504	2
505	2
506	3
507	3
508	2
509	2
510	2

468	0
469	4
470	0
471	0
472	2
473	1
474	0
475	1
476	0
477	2
478	2
479	0
480	1
481	0
482	1
483	4
484	4
485	0
486	0
487	2
488	0
489	3
490	2
491	1
492	1
493	1
494	1
495	5
496	4
497	0
498	2
499	1
500	1
501	5
502	3
503	1
504	3
505	4
506	5
507	2
508	0
509	2
510	0

468	4
469	0
470	1
471	0
472	4
473	3
474	3
475	0
476	0
477	5
478	3
479	2
480	3
481	4
482	0
483	2
484	1
485	0
486	2
487	0
488	3
489	3
490	0
491	0
492	0
493	2
494	0
495	0
496	0
497	4
498	3
499	0
500	0
501	2
502	1
503	4
504	1
505	0
506	1
507	1
508	4
509	3
510	0

511	3
512	3
513	1
514	1
515	4
516	2
517	0
518	1
519	0
520	4
521	3
522	1
523	4
524	3
525	0
526	2
527	3
528	3
529	4
530	2
531	2
532	1
533	1
534	2
535	3
536	2
537	3
538	2
539	3
540	6
541	2
542	3
543	1
544	3
545	1
546	1
547	3
548	2
549	0
550	2
551	1
552	2
553	1

511	2
512	2
513	3
514	3
515	3
516	4
517	2
518	2
519	0
520	0
521	1
522	1
523	0
524	3
525	1
526	3
527	2
528	3
529	3
530	3
531	2
532	3
533	2
534	3
535	1
536	3
537	5
538	5
539	0
540	3
541	3
542	2
543	0
544	1
545	2
546	2
547	2
548	0
549	3
550	2
551	4
552	4
553	2

511	0
512	3
513	2
514	0
515	2
516	1
517	1
518	3
519	3
520	4
521	3
522	4
523	2
524	3
525	3
526	3
527	3
528	3
529	1
530	4
531	4
532	3
533	3
534	3
535	5
536	5
537	5
538	0
539	5
540	3
541	0
542	3
543	0
544	4
545	5
546	3
547	4
548	5
549	2
550	3
551	0
552	5
553	3

554	2
555	2
556	2
557	1
558	3
559	1
560	4
561	3
562	1
563	3
564	1
565	2
566	2
567	0
568	5
569	2
570	1
571	3
572	3
573	1
574	3
575	1
576	1
577	3
578	3
579	1
580	3
581	3
582	3
583	3
584	2
585	2
586	3
587	1
588	2
589	3
590	3
591	3
592	2
593	3
594	1
595	2
596	1

554	0
555	4
556	0
557	3
558	0
559	2
560	4
561	3
562	4
563	0
564	1
565	4
566	2
567	3
568	0
569	1
570	2
571	0
572	2
573	1
574	1
575	1
576	2
577	2
578	2
579	2
580	3
581	0
582	3
583	2
584	4
585	0
586	3
587	4
588	2
589	2
590	0
591	3
592	3
593	2
594	3
595	3
596	1

554	2
555	3
556	0
557	0
558	0
559	2
560	5
561	2
562	2
563	3
564	0
565	5
566	0
567	3
568	0
569	5
570	1
571	0
572	1
573	4
574	0
575	0
576	4
577	4
578	4
579	0
580	0
581	3
582	0
583	0
584	0
585	3
586	1
587	0
588	0
589	3
590	0
591	3
592	1
593	0
594	1
595	0
596	0

597	2
598	3
599	3
600	0
601	4
602	3
603	2
604	2
605	2
606	1
607	3
608	1
609	2
610	3
611	2
612	3
613	3
614	2
615	3
616	2
617	1
618	3
619	4
620	2
621	3
622	2
623	1
624	2
625	3
626	0
627	3
628	2
629	2
630	1
631	3
632	2
633	1
634	2
635	0
636	1
637	1
638	2
639	0

597	5
598	2
599	5
600	1
601	4
602	0
603	1
604	3
605	1
606	0
607	3
608	2
609	4
610	3
611	1
612	4
613	2
614	0
615	1
616	3
617	0
618	2
619	4
620	2
621	3
622	5
623	3
624	3
625	0
626	0
627	4
628	3
629	3
630	4
631	3
632	2
633	2
634	1
635	3
636	2
637	3
638	4
639	4

597	3
598	0
599	3
600	1
601	5
602	4
603	4
604	3
605	2
606	2
607	0
608	3
609	0
610	3
611	3
612	0
613	3
614	2
615	1
616	3
617	2
618	3
619	3
620	3
621	2
622	0
623	0
624	2
625	4
626	3
627	0
628	3
629	0
630	5
631	3
632	0
633	0
634	3
635	0
636	5
637	3
638	1
639	2

640	1
641	1
642	1
643	2
644	2
645	0
646	2
647	1
648	1
649	0
650	2
651	1
652	3
653	1
654	3
655	1
656	2
657	1
658	2
659	1
660	2
661	2
662	2
663	3
664	2
665	2
666	1
667	3
668	1
669	1
670	3
671	2
672	2
673	2
674	3
675	1
676	3
677	3
678	1
679	3
680	2
681	0
682	3

640	1
641	1
642	5
643	3
644	4
645	2
646	3
647	0
648	5
649	0
650	0
651	1
652	3
653	2
654	3
655	3
656	0
657	4
658	3
659	1
660	3
661	2
662	2
663	4
664	0
665	2
666	5
667	0
668	2
669	2
670	3
671	4
672	3
673	2
674	3
675	4
676	0
677	3
678	0
679	2
680	1
681	2
682	2

640	0
641	0
642	2
643	3
644	2
645	2
646	2
647	0
648	1
649	3
650	5
651	0
652	0
653	0
654	0
655	0
656	2
657	0
658	0
659	0
660	3
661	0
662	1
663	2
664	0
665	0
666	2
667	4
668	2
669	2
670	3
671	4
672	0
673	3
674	1
675	0
676	0
677	0
678	2
679	2
680	3
681	2
682	4

683	1
684	3
685	0
686	2
687	1
688	2
689	1
690	3
691	2
692	2
693	2
694	1
695	1
696	2
697	3
698	3
699	2
700	0
701	1
702	1
703	2
704	1
705	2
706	3
707	3
708	3
709	2
710	1
711	2
712	2
713	0
714	2
715	1
716	2
717	2
718	2
719	1
720	2
721	1
722	3
723	2
724	3
725	3

683	1
684	4
685	3
686	3
687	2
688	1
689	2
690	0
691	0
692	0
693	0
694	0
695	0
696	4
697	5
698	3
699	2
700	4
701	2
702	0
703	0
704	0
705	1
706	3
707	5
708	0
709	3
710	0
711	0
712	2
713	0
714	0
715	1
716	2
717	4
718	2
719	3
720	0
721	0
722	3
723	0
724	0
725	3

683	0
684	0
685	3
686	0
687	0
688	3
689	3
690	3
691	4
692	3
693	3
694	3
695	2
696	5
697	0
698	4
699	3
700	4
701	5
702	1
703	0
704	0
705	4
706	3
707	4
708	3
709	3
710	4
711	0
712	0
713	5
714	2
715	5
716	0
717	2
718	0
719	0
720	0
721	3
722	0
723	0
724	0
725	0

726	3
727	3
728	3
729	3
730	1
731	3
732	2
733	3
734	2
735	2
736	4
737	2
738	4
739	2
740	3
741	2
742	3
743	0
744	2
745	3
746	1
747	2
748	4
749	2
750	2
751	1
752	2
753	3
754	2
755	2
756	2
757	1
758	2
759	1
760	0
761	4
762	3
763	1
764	0
765	3
766	3
767	1
768	1

726	3
727	1
728	2
729	4
730	0
731	0
732	2
733	0
734	0
735	3
736	3
737	2
738	3
739	4
740	4
741	1
742	0
743	0
744	0
745	1
746	3
747	3
748	0
749	4
750	2
751	3
752	1
753	4
754	1
755	3
756	2
757	4
758	3
759	2
760	1
761	4
762	1
763	0
764	4
765	1
766	4
767	0
768	3

726	0
727	0
728	3
729	0
730	0
731	4
732	1
733	4
734	0
735	4
736	0
737	3
738	2
739	3
740	4
741	0
742	4
743	0
744	0
745	2
746	0
747	2
748	3
749	0
750	0
751	3
752	5
753	0
754	3
755	4
756	5
757	4
758	0
759	3
760	3
761	4
762	3
763	4
764	2
765	0
766	5
767	3
768	1

769	2
770	1
771	2
772	2
773	1
774	2
775	4
776	3
777	3
778	3
779	3
780	3
781	4
782	1
783	1
784	3
785	4
786	3
787	2
788	3
789	0
790	0
791	3
792	3
793	2
794	3
795	3
796	4
797	2
798	3
799	1
800	2
801	3
802	2
803	1
804	3
805	1
806	2
807	1
808	3
809	3
810	1
811	1

769	3
770	1
771	2
772	2
773	0
774	4
775	3
776	1
777	3
778	3
779	3
780	2
781	3
782	4
783	2
784	1
785	2
786	5
787	4
788	3
789	2
790	4
791	2
792	2
793	3
794	4
795	2
796	1
797	0
798	4
799	2
800	0
801	4
802	5
803	3
804	5
805	1
806	1
807	3
808	3
809	2
810	0
811	0

769	3
770	5
771	2
772	0
773	3
774	3
775	0
776	0
777	1
778	1
779	1
780	5
781	3
782	1
783	1
784	2
785	1
786	0
787	0
788	4
789	2
790	0
791	3
792	3
793	4
794	1
795	5
796	4
797	4
798	1
799	0
800	1
801	1
802	5
803	0
804	0
805	3
806	5
807	1
808	0
809	2
810	2
811	0

812	1
813	3
814	1
815	3
816	2
817	1
818	1
819	2
820	3
821	1
822	2
823	1
824	2
825	1
826	2
827	3
828	3
829	2
830	3
831	3
832	4
833	1
834	3
835	3
836	1
837	3
838	3
839	1
840	3
841	1
842	3
843	2
844	2
845	2
846	1
847	2
848	2
849	3
850	2
851	3
852	3
853	3
854	3

812	3
813	1
814	2
815	4
816	0
817	1
818	3
819	5
820	4
821	3
822	0
823	0
824	1
825	0
826	4
827	4
828	1
829	1
830	4
831	2
832	2
833	0
834	1
835	2
836	0
837	3
838	0
839	1
840	2
841	4
842	4
843	5
844	4
845	3
846	3
847	3
848	0
849	1
850	4
851	5
852	2
853	5
854	2

812	0
813	3
814	2
815	3
816	3
817	4
818	2
819	5
820	2
821	2
822	3
823	5
824	1
825	1
826	4
827	3
828	2
829	0
830	3
831	5
832	2
833	2
834	2
835	5
836	2
837	3
838	2
839	2
840	2
841	4
842	4
843	0
844	0
845	5
846	0
847	1
848	4
849	4
850	2
851	2
852	2
853	0
854	0

855	1
856	1
857	1
858	3
859	1
860	4
861	4
862	1
863	1
864	2
865	1
866	3
867	2
868	2
869	0
870	2
871	3
872	3
873	2
874	1
875	2
876	3
877	3
878	2
879	3
880	4
881	2
882	4
883	2
884	3
885	2
886	2
887	2
888	3
889	3
890	1
891	3
892	3
893	2
894	1
895	4
896	1
897	2

855	1
856	1
857	3
858	3
859	2
860	1
861	5
862	3
863	3
864	3
865	3
866	0
867	3
868	0
869	4
870	3
871	0
872	2
873	2
874	1
875	1
876	3
877	2
878	3
879	0
880	4
881	5
882	3
883	3
884	4
885	1
886	4
887	2
888	4
889	2
890	3
891	5
892	1
893	3
894	1
895	3
896	3
897	2

855	4
856	0
857	0
858	1
859	1
860	2
861	1
862	0
863	1
864	1
865	4
866	2
867	1
868	2
869	3
870	0
871	3
872	3
873	3
874	2
875	2
876	0
877	0
878	0
879	2
880	1
881	0
882	1
883	4
884	0
885	2
886	0
887	0
888	0
889	0
890	0
891	0
892	2
893	2
894	1
895	0
896	0
897	0

898	2
899	2
900	3
901	4
902	4
903	0
904	3
905	3
906	1
907	0
908	3
909	3
910	3
911	2
912	2
913	2
914	1
915	4
916	1
917	4
918	3
919	3
920	1
921	1
922	1
923	2
924	1
925	7
926	1
927	3
928	2
929	0
930	1
931	3
932	1
933	1
934	2
935	3
936	3
937	2
938	2
939	3
940	3

898	4
899	2
900	2
901	3
902	2
903	3
904	1
905	4
906	0
907	0
908	5
909	2
910	2
911	1
912	3
913	1
914	1
915	1
916	3
917	0
918	4
919	5
920	3
921	5
922	4
923	2
924	1
925	4
926	0
927	2
928	2
929	3
930	3
931	5
932	2
933	2
934	2
935	4
936	1
937	0
938	4
939	5
940	2

898	4
899	0
900	2
901	2
902	0
903	0
904	0
905	0
906	4
907	0
908	1
909	1
910	2
911	3
912	0
913	0
914	4
915	0
916	0
917	2
918	1
919	0
920	2
921	1
922	3
923	2
924	1
925	3
926	3
927	2
928	1
929	2
930	2
931	2
932	4
933	1
934	0
935	0
936	1
937	1
938	0
939	0
940	0

941	3
942	3
943	4
944	3
945	2
946	2
947	0
948	1
949	2
950	1
951	2
952	2
953	2
954	3
955	2
956	3
957	3
958	3
959	3
960	2
961	1
962	1
963	4
964	0
965	3
966	2
967	2
968	2
969	3
970	2
971	2
972	2
973	2
974	1
975	3
976	1
977	3
978	2
979	1
980	2
981	2
982	1
983	2

941	1
942	1
943	3
944	3
945	0
946	2
947	2
948	3
949	4
950	2
951	4
952	1
953	3
954	1
955	2
956	2
957	3
958	2
959	0
960	0
961	0
962	4
963	3
964	3
965	2
966	1
967	2
968	3
969	1
970	1
971	1
972	0
973	0
974	2
975	1
976	5
977	2
978	3
979	3
980	1
981	2
982	1
983	3

941	1
942	0
943	1
944	1
945	2
946	0
947	0
948	0
949	3
950	3
951	4
952	1
953	0
954	2
955	0
956	0
957	1
958	1
959	0
960	4
961	0
962	1
963	2
964	3
965	0
966	2
967	0
968	0
969	2
970	0
971	0
972	0
973	0
974	2
975	0
976	0
977	3
978	2
979	0
980	1
981	3
982	2
983	3

984	3
985	3
986	2
987	3
988	1
989	2
990	2
991	2
992	1
993	4
994	3
995	2
996	4
997	2
998	1
999	2
1000	2
1001	4

984	1
985	3
986	4
987	0
988	2
989	4
990	1
991	1
992	2
993	1
994	2
995	4
996	1
997	1
998	2
999	2
1000	0
1001	4
1002	2
1003	0
1004	4
1005	0
1006	4
1007	2
1008	3
1009	3
1010	2
1011	2
1012	0
1013	0
1014	2
1015	2
1016	3
1017	3
1018	0
1019	3
1020	1
1021	1
1022	3
1023	2
1024	2
1025	0
1026	1

984	3
985	2
986	0
987	1
988	1
989	1
990	1
991	2
992	2
993	2
994	2
995	0
996	1
997	2
998	1
999	2
1000	0
1001	2
1002	4
1003	0
1004	5
1005	0
1006	3
1007	3
1008	1
1009	1
1010	3
1011	1
1012	0
1013	2
1014	2
1015	0
1016	2
1017	4
1018	2
1019	3
1020	2
1021	4
1022	2
1023	5
1024	0
1025	4
1026	3

1027	1
1028	3
1029	5
1030	0
1031	1
1032	2
1033	3
1034	0
1035	0
1036	4
1037	3
1038	0
1039	3
1040	3
1041	4
1042	0
1043	0
1044	1
1045	0
1046	3
1047	0
1048	1
1049	3
1050	0
1051	5
1052	4
1053	2
1054	3
1055	1
1056	3
1057	2
1058	2
1059	2
1060	0
1061	2
1062	1
1063	0
1064	1
1065	2
1066	3
1067	1
1068	0
1069	2

1027	5
1028	4
1029	0
1030	2
1031	5
1032	3
1033	3
1034	4
1035	3
1036	3
1037	5
1038	4
1039	3
1040	3
1041	3
1042	5
1043	4
1044	0
1045	0
1046	3
1047	0
1048	3
1049	3
1050	5
1051	2
1052	0
1053	0
1054	0
1055	4
1056	2
1057	5
1058	5
1059	5
1060	4
1061	0
1062	4
1063	1
1064	0
1065	2
1066	0
1067	5
1068	0
1069	2

1070	4
1071	2
1072	4
1073	3
1074	0
1075	3
1076	4
1077	4
1078	4
1079	2
1080	3
1081	2
1082	2
1083	4
1084	4
1085	2
1086	2
1087	2
1088	3
1089	4
1090	3
1091	3
1092	5
1093	1
1094	4
1095	1
1096	3
1097	0
1098	0
1099	3
1100	1
1101	4
1102	4
1103	0
1104	3
1105	4
1106	1
1107	0
1108	1
1109	3
1110	5
1111	0
1112	4

1070	0
1071	4
1072	0
1073	0
1074	0
1075	4
1076	3
1077	4
1078	3
1079	1
1080	0
1081	3
1082	2
1083	3
1084	2
1085	3
1086	3
1087	0
1088	1
1089	2
1090	4
1091	1
1092	2
1093	5
1094	5
1095	4
1096	0
1097	2
1098	1
1099	4
1100	4
1101	3
1102	2
1103	5
1104	0
1105	2
1106	0
1107	0
1108	5
1109	4
1110	5
1111	0
1112	0

1113	3
1114	3
1115	2
1116	0
1117	0
1118	5
1119	2
1120	3
1121	1
1122	0
1123	3
1124	2
1125	3
1126	3
1127	3
1128	4
1129	0
1130	4
1131	3
1132	3
1133	3
1134	4
1135	0
1136	2
1137	4
1138	4
1139	2
1140	0
1141	0
1142	0
1143	4
1144	2
1145	3
1146	1
1147	2
1148	2
1149	2
1150	5
1151	3
1152	2
1153	4
1154	2
1155	4

1113	3
1114	0
1115	0
1116	4
1117	2
1118	4
1119	0
1120	0
1121	0
1122	0
1123	1
1124	0
1125	6
1126	0
1127	4
1128	3
1129	2
1130	4
1131	0
1132	0
1133	3
1134	3
1135	3
1136	0
1137	1
1138	3
1139	4
1140	2
1141	2
1142	0
1143	0
1144	0
1145	0
1146	3
1147	0
1148	4
1149	4
1150	4
1151	5
1152	4
1153	4
1154	3
1155	2

1156	1
1157	1
1158	3
1159	2
1160	2
1161	3
1162	2
1163	4
1164	0
1165	0
1166	4
1167	3
1168	2
1169	2
1170	1
1171	3
1172	2
1173	0
1174	0
1175	4
1176	3
1177	2
1178	4
1179	2
1180	1
1181	3
1182	3
1183	3
1184	0
1185	3
1186	2
1187	0
1188	3
1189	1
1190	4
1191	2
1192	5
1193	2
1194	0
1195	3
1196	3
1197	2
1198	0

1156	0
1157	4
1158	3
1159	2
1160	0
1161	5
1162	0
1163	3
1164	3
1165	3
1166	0
1167	2
1168	0
1169	4
1170	0
1171	0
1172	0
1173	3
1174	3
1175	2
1176	0
1177	4
1178	2
1179	0
1180	4
1181	0
1182	0
1183	4
1184	0
1185	3
1186	3
1187	1
1188	0
1189	1
1190	0
1191	0
1192	0
1193	3
1194	3
1195	0
1196	0
1197	0
1198	0

1199	3
1200	2
1201	3
1202	4
1203	4
1204	4
1205	5
1206	2
1207	4
1208	2
1209	2
1210	3
1211	4
1212	1
1213	2
1214	4
1215	1
1216	5
1217	4
1218	1
1219	2
1220	0
1221	2
1222	4
1223	2
1224	1
1225	4
1226	4
1227	5
1228	2
1229	2
1230	3
1231	1
1232	2
1233	4
1234	1
1235	3
1236	3
1237	0
1238	2
1239	3
1240	3
1241	1

1199	0
1200	0
1201	0
1202	1
1203	1
1204	2
1205	2
1206	1
1207	0
1208	3
1209	0
1210	2
1211	2
1212	1
1213	0
1214	1
1215	0
1216	0
1217	0
1218	4
1219	0
1220	3
1221	0
1222	2
1223	2
1224	4
1225	3
1226	0
1227	3
1228	3
1229	3
1230	4
1231	3
1232	3
1233	2
1234	3
1235	2
1236	3
1237	3
1238	3
1239	0
1240	4
1241	2

1242	2
1243	2
1244	3
1245	1
1246	3
1247	4
1248	2
1249	4
1250	4
1251	3
1252	4
1253	1
1254	4
1255	1
1256	3
1257	3
1258	2
1259	2
1260	4
1261	1
1262	5
1263	4
1264	1
1265	0
1266	3
1267	0
1268	3
1269	3
1270	0
1271	4
1272	0
1273	4
1274	2
1275	4
1276	3
1277	5
1278	0
1279	1
1280	2
1281	6
1282	5
1283	0
1284	0

1242	4
1243	2
1244	4
1245	3
1246	2
1247	3
1248	3
1249	0
1250	2
1251	0
1252	2
1253	0
1254	2
1255	0
1256	4
1257	3
1258	4
1259	2
1260	3
1261	3
1262	0
1263	5
1264	3
1265	4
1266	4
1267	4
1268	0
1269	4
1270	4
1271	3
1272	4
1273	3
1274	2
1275	2
1276	5
1277	5
1278	1
1279	2
1280	3
1281	2
1282	4
1283	4
1284	4

1285	0
1286	1
1287	0
1288	5
1289	4
1290	0
1291	1
1292	5
1293	3
1294	0
1295	1
1296	1
1297	4
1298	3
1299	0
1300	4
1301	4
1302	4
1303	0
1304	2
1305	2
1306	2
1307	3
1308	4
1309	0
1310	4
1311	0
1312	3
1313	2
1314	1
1315	5
1316	2
1317	3
1318	0
1319	3
1320	2
1321	0
1322	2
1323	0
1324	3
1325	3
1326	3
1327	0

1285	4
1286	3
1287	2
1288	2
1289	3
1290	4
1291	3
1292	1
1293	0
1294	2
1295	5
1296	4
1297	3
1298	3
1299	2
1300	1
1301	2
1302	4
1303	0
1304	4
1305	2
1306	3
1307	3
1308	2
1309	3
1310	4
1311	4
1312	3
1313	4
1314	4
1315	4
1316	0
1317	0
1318	5
1319	1
1320	2
1321	2
1322	4
1323	0
1324	0
1325	3
1326	3
1327	0

1328	0
1329	0
1330	4
1331	3
1332	3
1333	4
1334	3
1335	3
1336	0
1337	3
1338	2
1339	3
1340	1
1341	1
1342	4
1343	3
1344	3
1345	4
1346	1
1347	3
1348	3
1349	3
1350	3
1351	1
1352	3
1353	3
1354	3
1355	1
1356	4
1357	1
1358	2
1359	0
1360	3
1361	3
1362	3
1363	5
1364	0
1365	4
1366	3
1367	0
1368	2
1369	0
1370	2

1328	3
1329	0
1330	2
1331	0
1332	4
1333	0
1334	1
1335	2
1336	3
1337	0
1338	3
1339	0
1340	0
1341	4
1342	3
1343	1
1344	0
1345	0
1346	2
1347	3
1348	4
1349	5
1350	0
1351	2
1352	0
1353	5
1354	5
1355	4
1356	4
1357	3
1358	3
1359	4
1360	5
1361	4
1362	5
1363	4
1364	5
1365	4
1366	5
1367	2
1368	2
1369	3
1370	3

1371	0
1372	4
1373	0
1374	3
1375	1
1376	2
1377	3
1378	0
1379	4
1380	2
1381	2
1382	3
1383	1
1384	2
1385	1
1386	5
1387	4
1388	4
1389	3
1390	2
1391	3
1392	5
1393	0
1394	1
1395	0
1396	1
1397	4
1398	2
1399	2
1400	3
1401	3
1402	3
1403	2
1404	0
1405	3
1406	0
1407	0
1408	4
1409	3
1410	5
1411	5
1412	5
1413	4

1371	4
1372	4
1373	3
1374	1
1375	5
1376	4
1377	4
1378	5
1379	5
1380	2
1381	2
1382	2
1383	3
1384	2
1385	3
1386	2
1387	5
1388	2
1389	0
1390	3
1391	2
1392	2
1393	0
1394	5
1395	2
1396	2
1397	4
1398	1
1399	4
1400	2
1401	0
1402	1
1403	0
1404	3
1405	2
1406	1
1407	3
1408	3
1409	0
1410	2
1411	0
1412	3
1413	1

1414	1
1415	1
1416	0
1417	1
1418	3
1419	1
1420	1
1421	4
1422	2
1423	3
1424	5
1425	4
1426	3
1427	4
1428	3
1429	4
1430	4
1431	4
1432	4
1433	3
1434	0
1435	2
1436	3
1437	0
1438	0
1439	2
1440	2
1441	4
1442	5
1443	3
1444	4
1445	1
1446	2
1447	1
1448	1
1449	2
1450	2
1451	5
1452	4
1453	1
1454	1
1455	4
1456	5

1414	3
1415	0
1416	2
1417	4
1418	4
1419	0
1420	3
1421	5
1422	1
1423	0
1424	2
1425	0
1426	4
1427	2
1428	5
1429	4
1430	4
1431	2
1432	5
1433	0
1434	1
1435	3
1436	2
1437	4
1438	2
1439	3
1440	3
1441	0
1442	0
1443	0
1444	1
1445	2
1446	1
1447	0
1448	0
1449	0
1450	4
1451	1
1452	1
1453	2
1454	3
1455	3
1456	2

1457	0
1458	5
1459	1
1460	4
1461	4
1462	0
1463	2
1464	0
1465	2
1466	4
1467	2
1468	0
1469	2
1470	2
1471	3
1472	4
1473	4
1474	2
1475	0
1476	3
1477	0
1478	1
1479	3
1480	2
1481	4
1482	4
1483	2
1484	3
1485	1
1486	1
1487	5
1488	2
1489	2
1490	2
1491	2
1492	5
1493	2
1494	1
1495	4
1496	1
1497	4
1498	0
1499	0

1457	2
1458	2
1459	0
1460	4
1461	0
1462	4
1463	4
1464	3
1465	2
1466	4
1467	1
1468	2
1469	1
1470	3
1471	2
1472	3
1473	4
1474	4
1475	4
1476	2
1477	4
1478	2
1479	4
1480	3
1481	1
1482	2
1483	2
1484	3
1485	4
1486	3
1487	0
1488	3
1489	2
1490	2
1491	1
1492	2
1493	3
1494	3
1495	0
1496	3
1497	3
1498	3
1499	0

1500	3
1501	4
1502	0
1503	3
1504	0
1505	2
1506	2
1507	4
1508	3
1509	5
1510	4
1511	1
1512	1
1513	2
1514	0
1515	3
1516	1
1517	4
1518	4
1519	0
1520	1
1521	2
1522	3
1523	1
1524	3
1525	1
1526	1
1527	2
1528	2
1529	4
1530	4
1531	0
1532	4
1533	2
1534	2
1535	3
1536	0
1537	4
1538	2
1539	4
1540	2
1541	3
1542	1

1500	0
1501	3
1502	1
1503	1
1504	1
1505	0
1506	2
1507	3
1508	0
1509	0
1510	0
1511	0
1512	0
1513	2
1514	3
1515	3
1516	0
1517	2
1518	2
1519	3
1520	4
1521	0
1522	3
1523	0
1524	3
1525	3
1526	3
1527	2
1528	0
1529	0
1530	0
1531	4
1532	3
1533	0
1534	5
1535	3
1536	4
1537	3
1538	5
1539	3
1540	3
1541	2
1542	0

1543	3
1544	2
1545	3
1546	2
1547	2
1548	1
1549	2
1550	3
1551	0
1552	4
1553	0
1554	0
1555	1
1556	5
1557	2
1558	2
1559	3
1560	1
1561	2
1562	4
1563	4
1564	3
1565	5
1566	3
1567	1
1568	2
1569	4
1570	5
1571	3
1572	2
1573	2
1574	2
1575	4
1576	1
1577	4
1578	4
1579	3
1580	3
1581	5
1582	3
1583	3
1584	3
1585	5

1543	4
1544	1
1545	2
1546	2
1547	3
1548	3
1549	0
1550	2
1551	5
1552	2
1553	4
1554	2
1555	4
1556	4
1557	2
1558	2
1559	2
1560	3
1561	0
1562	4
1563	3
1564	1
1565	0
1566	2
1567	2
1568	4
1569	3
1570	2
1571	1
1572	4
1573	1
1574	1
1575	3
1576	3
1577	3
1578	1
1579	0
1580	1
1581	0
1582	4
1583	3
1584	6
1585	5

1586	0
1587	2
1588	0
1589	0
1590	1
1591	0
1592	1
1593	4
1594	3
1595	1
1596	1
1597	1
1598	1
1599	2
1600	5
1601	2
1602	2
1603	2
1604	3
1605	5
1606	4
1607	1
1608	2
1609	0
1610	2
1611	1
1612	3
1613	1
1614	0
1615	2
1616	2
1617	3
1618	4
1619	3
1620	0
1621	0
1622	4
1623	1
1624	3
1625	0
1626	3
1627	0
1628	3

1586	4
1587	3
1588	2
1589	0
1590	1
1591	3
1592	5
1593	3
1594	3
1595	2
1596	0
1597	4
1598	3
1599	4
1600	2
1601	5
1602	4
1603	3
1604	4
1605	5
1606	5
1607	2
1608	2
1609	4
1610	4
1611	5
1612	3
1613	3
1614	5
1615	2
1616	3
1617	3
1618	2
1619	4
1620	2
1621	2
1622	3
1623	3
1624	1
1625	1
1626	3
1627	2
1628	1

1629	5
1630	0
1631	4
1632	5
1633	2
1634	1
1635	4
1636	3
1637	0
1638	0
1639	1
1640	0
1641	3
1642	0
1643	0
1644	0
1645	4
1646	4
1647	3
1648	3
1649	4
1650	0
1651	0
1652	3
1653	3
1654	1
1655	5
1656	5
1657	3
1658	3
1659	3
1660	0
1661	3
1662	3
1663	2
1664	4
1665	5
1666	0
1667	2
1668	1
1669	4
1670	3
1671	0

1629	4
1630	2
1631	0
1632	3
1633	5
1634	5
1635	3
1636	1
1637	1
1638	2
1639	4
1640	1
1641	4
1642	2
1643	5
1644	4
1645	4
1646	2
1647	4
1648	1
1649	4
1650	4
1651	4
1652	5
1653	2
1654	1
1655	1
1656	3
1657	3
1658	0
1659	2
1660	4
1661	0
1662	0
1663	3
1664	4
1665	0
1666	2
1667	2
1668	3
1669	0
1670	1
1671	1

1672	2
1673	1
1674	4
1675	2
1676	4
1677	0
1678	2
1679	3
1680	3
1681	2
1682	0
1683	3
1684	1
1685	2
1686	5
1687	5
1688	2
1689	2
1690	4
1691	2
1692	3
1693	4
1694	1
1695	1
1696	4
1697	3
1698	2
1699	2
1700	1
1701	5
1702	4
1703	4
1704	2
1705	2
1706	0
1707	0
1708	1
1709	2
1710	4
1711	4
1712	2
1713	3
1714	4

1672	1
1673	2
1674	3
1675	4
1676	0
1677	1
1678	0
1679	5
1680	2
1681	4
1682	3
1683	5
1684	3
1685	1
1686	4
1687	3
1688	5
1689	3
1690	1
1691	1
1692	3
1693	1
1694	2
1695	4
1696	2
1697	2
1698	4
1699	4
1700	4
1701	1
1702	4
1703	3
1704	5
1705	4
1706	2
1707	4
1708	4
1709	3
1710	2
1711	4
1712	3
1713	4
1714	4

1715	2
1716	2
1717	2
1718	3
1719	0
1720	2
1721	3
1722	5
1723	1

1715	2
1716	2
1717	5
1718	0
1719	4
1720	4
1721	0
1722	0
1723	4
1724	3
1725	3
1726	0
1727	0
1728	0
1729	1
1730	3
1731	2
1732	3
1733	4
1734	3
1735	3
1736	3
1737	4
1738	0
1739	5
1740	0
1741	3
1742	0
1743	1
1744	3
1745	3
1746	3
1747	3
1748	2
1749	5
1750	3
1751	5
1752	3
1753	3
1754	5
1755	1
1756	3
1757	2

1758	3
1759	1
1760	1
1761	5
1762	5
1763	2
1764	4
1765	5
1766	2
1767	2
1768	5
1769	3
1770	4
1771	1
1772	2
1773	2
1774	0
1775	4
1776	1
1777	0
1778	5
1779	4
1780	4
1781	3
1782	3
1783	5
1784	4
1785	3
1786	1
1787	5
1788	3
1789	5
1790	3
1791	4
1792	3
1793	3
1794	2
1795	3
1796	5
1797	1
1798	4
1799	2
1800	3

1801	0
1802	0
1803	4
1804	2
1805	5
1806	2
1807	4
1808	0
1809	4
1810	1
1811	5
1812	1
1813	2
1814	3
1815	2
1816	1
1817	4
1818	1
1819	3
1820	0
1821	5
1822	0
1823	4
1824	2
1825	3
1826	4
1827	0
1828	4
1829	3
1830	2
1831	4
1832	2
1833	2
1834	2
1835	5
1836	3
1837	1
1838	2
1839	3
1840	3
1841	0
1842	2
1843	3

1844	3
1845	2
1846	0
1847	4
1848	0
1849	3
1850	3
1851	0
1852	0
1853	0
1854	0
1855	0
1856	1
1857	2
1858	3
1859	3
1860	4
1861	3
1862	4
1863	4
1864	4
1865	3
1866	4
1867	0
1868	4
1869	4
1870	5
1871	3
1872	2
1873	2
1874	4
1875	4
1876	2
1877	4
1878	4
1879	5
1880	3
1881	0
1882	2
1883	1
1884	1
1885	4
1886	3

1887	4
1888	1
1889	3
1890	4
1891	5
1892	4
1893	1
1894	5
1895	4
1896	4
1897	2
1898	3
1899	4
1900	2
1901	1
1902	0
1903	0
1904	4
1905	1
1906	0
1907	0
1908	4
1909	3
1910	4
1911	4
1912	3
1913	5
1914	4
1915	4
1916	0
1917	4
1918	1
1919	4
1920	3
1921	6
1922	4
1923	4
1924	2
1925	2
1926	2
1927	3
1928	1
1929	5

1930	1
1931	4
1932	3
1933	5
1934	0
1935	3
1936	1
1937	5
1938	2
1939	3
1940	3
1941	1
1942	5
1943	2
1944	2
1945	5
1946	4
1947	2
1948	2
1949	3
1950	5
1951	9
1952	4
1953	2
1954	2
1955	2
1956	3
1957	5
1958	5
1959	0
1960	3
1961	4
1962	0
1963	5
1964	2
1965	4
1966	2
1967	5
1968	5
1969	3
1970	2
1971	2
1972	2

1973	2
1974	0
1975	2
1976	5
1977	2
1978	4
1979	3
1980	2
1981	3
1982	2
1983	4
1984	4
1985	3
1986	3
1987	4
1988	1
1989	2
1990	0
1991	2
1992	1
1993	4
1994	3
1995	1
1996	5
1997	4
1998	1
1999	3
2000	4
2001	3
2002	4
2003	0
2004	6
2005	4
2006	3
2007	5
2008	5
2009	2
2010	4
2011	2
2012	1
2013	4
2014	4
2015	5

2016	5
2017	2
2018	4
2019	5
2020	3
2021	5
2022	5
2023	3
2024	5
2025	3
2026	5
2027	4
2028	1
2029	0
2030	5
2031	0
2032	0
2033	3
2034	4
2035	4
2036	4
2037	3
2038	1
2039	8
2040	0
2041	3
2042	5
2043	3
2044	5
2045	3
2046	4
2047	4
2048	4
2049	3
2050	1
2051	3
2052	3
2053	3
2054	2
2055	2
2056	3
2057	5
2058	4

2059	5
2060	3
2061	3
2062	5
2063	0
2064	0
2065	3
2066	5
2067	1
2068	3
2069	3
2070	3
2071	6
2072	1
2073	3
2074	4
2075	3
2076	1
2077	2
2078	4
2079	5
2080	3
2081	3
2082	4
2083	5
2084	0
2085	3
2086	4
2087	2
2088	1
2089	3
2090	3
2091	2
2092	1
2093	1
2094	4
2095	4
2096	1
2097	3
2098	2
2099	3
2100	1
2101	5

2102	5
2103	5
2104	2
2105	2
2106	3
2107	2
2108	5
2109	3
2110	0
2111	4
2112	3
2113	3
2114	3
2115	4
2116	4
2117	4
2118	4
2119	5
2120	5
2121	0
2122	4
2123	2
2124	4
2125	4
2126	2
2127	2
2128	2
2129	2
2130	2
2131	4
2132	3
2133	5
2134	2
2135	4
2136	2
2137	3
2138	2
2139	5
2140	4
2141	2
2142	4
2143	1
2144	2

2145	5
2146	1
2147	1
2148	5
2149	5
2150	2
2151	2
2152	3
2153	2
2154	4
2155	5
2156	5
2157	2
2158	2
2159	4
2160	3
2161	1
2162	4
2163	2
2164	2
2165	2
2166	1
2167	2
2168	2
2169	7
2170	4
