# Supplementary material for: PT-Flax (phenotyping and TILLinG of flax): development of a flax (Linum usitatissimum L.) mutant population and TILLinG platform for forward and reverse genetics
Source: BMC Plant Biol. 2013 Oct 15;13:159. doi: 10.1186/1471-2229-13-159 (PMC3853753; doi:10.1186/1471-2229-13-159)
Supplement: Additional file 3 — PSSM/Sift scores and phenotypes for CAD mutant lines. PSSM scores and Sift scores were obtained with PARSESNP and SIFT programs, respectively. Scores in bold text are predicted to be damaging for protein activity while scores in normal text are predicted to be without effect. Mutations with no scores result from the lack of alignment block for the given position. The intensity (0, 1, 2, 3) of the brown-midrib phenotype (orange coloured xylem) in different CAD mutant lines is indicated in the column 'phenotype class’ and corresponds to Figure 5. Class 0 corresponds to wt phenotype. ND = not determined. Numbers in brackets after mutation (first column) indicate the number of families for which a given mutation was independently found. The absence of a number in brackets indicates that the mutation was only found in a single family. [file 1471-2229-13-159-S3.pdf]

Supplementary data 3

Mutation	PSSM difference	Sift score	Category
A158T	11.0	0,09	1
A158V	3.2	0.59	ND
A159V [3]	7.4	0.20	2, 3, 3
P160S	**8.9**	0.41	2
G165E	**24.7**	0.27	2
V166M	7.8	0.14	0
G176E	-	-	2
G176R	-	-	1
Q179STOP	-	-	1
R183K	0.7	1.00	ND
G188R [3]	**30.4**	**0.00**	1, 3, 0
G190D	**31.1**	**0.00**	2
G191E	**12.0**	**0.01**	0
A200T	**10.9**	0.08	0
G204D	**16.2**	**0.01**	2
H205Q	8.0	**0.01**	1
H206Y	3.6	0.08	0
S213P	-2,8	0,32	0
S213F [4]	4.4	0.06	2, 2, 0, 2
A220T	5.5	0.32	2
E222K	-3,5	1.00	3
G225R	**12.8**	0.09	1
A226V	-	-	0
D243N	-	-	3
T251I	**11.4**	**0.04**	0
V252I	7.3	0.11	1
P253S	-7.2	0.07	3
P257S	-5.3	0.42	2
P280L	-	-	1
S285N	-	-	ND
P286L	-	-	2
A294T	-	-	ND
A294V	-	-	3

G297R	-	-	2
V300M	-	-	3
E308K	-	-	3
R314K [2]	-	-	2, 2
G317R [2]	-	-	2, 0
M321I	-	-	ND
V324M	-	-	0
E351K [2]	-	-	1, 3
